# Advancing precision immunotherapy in advanced pancreatic cancer: a systematic review and meta-analysis of first-line ICI-based combinations

**DOI:** 10.3389/fimmu.2026.1855859

**Published:** 2026-07-10

**Authors:** Xiaoqi Qi, Haoran Yang, Li Zhang, Peishi Yu, Can Cheng

**Affiliations:** 1 School of Pharmacy, Tianjin Medical University, Tianjin, China; 2 The Second Clinical Medical College, Tianjin Medical University, Tianjin, China; 3 Department of Dermatology, Shenzhen Qianhai Shekou Free Trade Zone Hospital, Shenzhen, China; 4 School of Medical Technology and Nursing, Shenzhen Polytechnic University, Shenzhen, China

**Keywords:** chemotherapy, combination therapy, efficacy, first-line treatment, immune checkpoint inhibitors, meta-analysis, pancreatic ductal adenocarcinoma, safety

## Abstract

**Objective:**

Pancreatic ductal adenocarcinoma (PDAC) has an extremely poor prognosis. Immune checkpoint inhibitor (ICI) monotherapy has shown limited efficacy in PDAC, whereas the potential clinical value of first-line ICI-based combination regimens remains unclear. Through a systematic review and meta-analysis, this study aimed to evaluate the efficacy and safety of first-line ICI-based combination regimens in advanced PDAC.

**Methods:**

PubMed, Embase, The Cochrane Library, Scopus, Web of Science, CNKI, Wanfang Data, VIP, and CBM were searched up to April 4, 2026, to identify clinical studies evaluating first-line ICI-based combination therapy for advanced PDAC. Study screening and data extraction were performed in accordance with PRISMA guidelines.

**Results:**

Eight clinical trials involving 379 patients were included. In RCT-only analyses, ICI-based combination regimens showed favorable but statistically non-definitive trends for OS (HR = 0.83, 95% CI: 0.65-1.06) and PFS (HR = 0.75, 95% CI: 0.53-1.05), while ORR was improved (OR = 2.19, 95% CI: 1.32-3.64). Single-arm pooled analysis showed a median PFS of 6.2 months and an ORR of 38.0%. In terms of safety, combination therapy increased the risk of specific Grade 3 or higher adverse events, but the overall incidence was clinically manageable.

**Conclusion:**

First-line ICI-based combination regimens showed encouraging antitumor activity in advanced PDAC, particularly for radiological response. However, definitive survival benefits were not established in RCT-only analyses, and further large-scale randomized trials are needed.

**Systematic review registration:**

https://www.crd.york.ac.uk/PROSPERO/view/CRD420261440306, identifier CRD420261440306.

## 1 Introduction

Pancreatic ductal adenocarcinoma (PDAC) is one of the most lethal malignancies of the digestive system, characterized by a global escalation in both incidence and mortality. According to the latest SEER database report, the 5-year survival rate for pancreatic cancer remains critically low at 12%, significantly lagging behind other common malignancies (1). Despite advancements in diagnostic precision and therapeutic interventions, patient prognosis remains poor. A multicenter study of 556 patients reported a median survival time of only 12.4 months and a 5-year survival rate of just 4.3% (2). Crucially, only 10%–20% of patients are candidates for surgical resection at diagnosis, while over 50% present with distant metastases, rendering systemic therapy the primary treatment modality for the majority (3).

In the systemic management of advanced PDAC, chemotherapy remains the cornerstone. While gemcitabine monotherapy was long considered the standard—yielding a median overall survival (mOS) of 6–8 months—modern intensive regimens such as gemcitabine plus albumin-bound paclitaxel (AG) or FOLFIRINOX have only modestly extended mOS to 8–11 months (4). SEER-based evidence suggests that integrating radiotherapy with chemotherapy may further extend mOS from 9 to 12 months, yet survival outcomes necessitate further optimization (5).

Recently, immune checkpoint inhibitors (ICIs), such as anti-PD-1/PD-L1 antibodies, have revolutionized the treatment of various cancers; however, their efficacy in PDAC remains suboptimal. This limited response is largely attributed to a profoundly immunosuppressive tumor microenvironment (TME), defined by sparse immune cell infiltration, dense fibrous stroma, and a high prevalence of tumor-associated macrophages (TAMs) and myeloid-derived suppressor cells (MDSCs) (6). Consequently, PDAC is often classified as a “cold tumor,” necessitating novel combination strategies to overcome therapeutic resistance (7). Although combining ICIs with chemotherapy aims to leverage immunogenic cell death (ICD) to enhance antitumor synergy, systematic evidence-based data regarding its clinical efficacy as a first-line treatment remain scarce and highly heterogeneous. Therefore, this systematic review and meta-analysis aim to evaluate the clinical efficacy and safety of ICI-chemotherapy combinations in advanced PDAC to summarize the current evidence, clarify its limitations, and inform the design of future randomized studies.

## 2 Methods

### 2.1 Literature search and selection criteria

The execution of this systematic review and meta-analysis strictly adhered to the PRISMA 2020 (Preferred Reporting Items for Systematic Reviews and Meta-Analyses) guidelines. To identify eligible studies, a comprehensive literature search was conducted across multiple electronic databases, including PubMed, Embase, The Cochrane Library, Scopus, Web of Science, CNKI, Wanfang Data, VIP, and the China Biomedical Literature Database (CBM), spanning from their respective inception dates to April 4, 2026. The search strategy utilized a strategic combination of Medical Subject Headings (MeSH) and free-text keywords, such as “Pancreatic Neoplasms,” “First-line Treatment,” “Immunotherapy,” “Immune Checkpoint Inhibitors,” “PD-1/PD-L1 Inhibitors,” and “Clinical Trial”.

The selection of studies was governed by a predefined PICO framework. The target population consisted of patients aged ≥18 years with pathologically confirmed advanced or metastatic pancreatic cancer (predominantly PDAC) who had received no prior systemic therapy. Eligible interventions involved treatment regimens utilizing immune checkpoint inhibitors (ICIs) in combination with chemotherapy, with or without adjunctive anti-angiogenic agents. Standard-of-care first-line chemotherapy served as the comparator in randomized controlled trials. Single-arm and non-randomized studies evaluating first-line ICI-based combination regimens were also eligible because the available RCT evidence in this field remains limited and these studies provide clinically relevant information on regimen diversity, feasibility, response patterns, and safety signals. However, their data were used only for descriptive synthesis of treatment regimens, response outcomes, and safety outcomes, and were not used for comparative effect-size pooling. Comparative effect-size pooling was restricted to randomized controlled trials with chemotherapy-controlled data. Regarding clinical outcomes, studies were required to report at least one of the primary clinical endpoints, namely overall survival (OS), progression-free survival (PFS), objective response rate (ORR), or safety data. Conversely, studies were excluded if they involved non-first-line settings, non-ICI-based immunotherapies, or control groups receiving radiotherapy. Furthermore, duplicate publications, studies with inaccessible full texts, those with insufficient or missing primary data, and trials lacking ethical approval or complete clinical registry information were systematically excluded.

### 2.2 Data extraction and quality assessment

Data extraction was performed independently by two investigators, with all discrepancies resolved through secondary review of the original literature, consensus-based discussion, or consultation with a senior third-party expert. The extracted data were categorized into four critical dimensions. Initially, study characteristics including the first author, publication year, DOI/PMID, geographic region, clinical trial registration number (NCT), and trial phase were recorded. Subsequently, patient baseline characteristics—comprising total sample size, sex distribution, median age, prevalence of liver metastases, and primary tumor location—were documented. The third dimension focused on efficacy endpoints, specifically median overall survival (mOS) and progression-free survival (mPFS) with corresponding 95% confidence intervals (CIs) and hazard ratios (HRs), as well as tumor response metrics like ORR, disease control rate (DCR), and duration of response (mDoR). Finally, safety endpoints were extracted using the number of treated patients as the denominator, focusing on Grade≥3 hematologic and non-hematologic toxicities, and the incidence of immune-related adverse events (irAEs) such as pneumonitis and hepatitis.

The methodological quality of the included literature was rigorously evaluated using standardized scales tailored to the respective study designs. Randomized controlled trials (RCTs) were assessed via the Jadad scale, focusing on randomization, allocation concealment, and attrition. Given the commonality of open-label designs in late-stage oncological trials, blinding items were conservatively assigned zero points, resulting in a modified total score of 7, where a score≥3 was predefined as high quality. For non-randomized or single-arm studies, the Methodological Index for Non-Randomized Studies (MINORS) was utilized. Eight core indicators—including the prospective nature of the design, the objectivity of endpoints, and the adequacy of follow-up—were scored on a 0–2 scale, with a maximum possible score of 16, ensuring a robust assessment of study validity.

### 2.3 Statistical analysis and evidence synthesis

All statistical analyses and graphical visualizations were performed using R software. Meta-analyses were conducted using the meta and metafor packages, and figures were generated using ggplot2 where appropriate. For time-to-event outcomes, including overall survival (OS) and progression-free survival (PFS), hazard ratios (HRs) and corresponding 95% confidence intervals (CIs) were extracted and pooled using the inverse-variance method. Comparative pooled HRs were calculated only for randomized controlled trials (RCTs), whereas non-randomized and single-arm studies were summarized descriptively and were not included in comparative effect-size pooling.

For dichotomous outcomes in head-to-head comparisons, including objective response rate (ORR), disease control rate (DCR), and adverse events, pooled odds ratios (ORs) or relative risks (RRs) with 95% CIs were calculated using the Mantel–Haenszel method. For single-arm proportions, including ORR and DCR, pooled estimates were calculated using either the inverse-variance method or a generalized linear mixed model (GLMM) with logit transformation to stabilize variances. Median overall survival (mOS) and median progression-free survival (mPFS) were reported with 95% CIs whenever available; when CIs were not reported or were not extractable from the original studies, the values were labeled as NR.

All effect sizes, I^2^ statistics, and P-values were reported to two decimal places, and P-values < 0.001 were reported as P < 0.001. Statistical heterogeneity was assessed using Cochran’s Q test and the I^2^ statistic. An I^2^ value <50% together with a Q-test P ≥ 0.10 was considered to indicate low heterogeneity, and a fixed-effect model was applied; otherwise, a random-effects model was used. Forest plots were generated to display pooled estimates, 95% CIs, I^2^ statistics, Cochran’s Q-test P-values, model type, subgroup interaction P-values, and sample sizes where applicable.

Sensitivity analyses were performed to evaluate the robustness of pooled estimates and the influence of individual studies. These analyses included leave-one-out analysis, RCT-only analysis, exclusion of non-randomized studies, exclusion of small-scale studies, comparison between fixed-effect and random-effects models, and sample-size stratification using a cutoff of 50 patients. Leave-one-out and exclusion-based analyses were conducted as robustness and influence diagnostics rather than procedures to identify statistically favorable results. Analyses added during revision in response to reviewer comments were treated as *post hoc* exploratory analyses rather than confirmatory analyses, and the primary conclusions were based on the prespecified main models. Publication bias was assessed using funnel plots, Egger’s linear regression test, and Begg’s rank correlation test when at least three studies were available; because of the limited number of included studies, these analyses were considered exploratory and were interpreted cautiously.

## 3 Results

### 3.1 Literature search and screening results

A systematic search across major international and domestic databases—including PubMed, Embase, The Cochrane Library, Scopus, and CNKI—yielded a total of 1,630 records. The initial distribution of these records was as follows: PubMed (n = 1,320, comprising 372 from the primary search and 948 from updated retrieval), Embase (n = 219), The Cochrane Library (n = 44), Scopus (n = 32), CNKI (n = 6), and 41 additional records identified through other sources (Wanfang, CBM, and VIP). Detailed search strategies and keyword constructions are provided in **Supplemetary Table 2**. After removing six duplicate records, 1,624 articles underwent title and abstract screening. Of these, 1,519 were excluded for irrelevance to the study theme, leaving 105 articles for full-text evaluation.

The flow of literature identification and selection is summarized in the PRISMA flowchart (**Supplementary Figure 1**). During the full-text review, 75 articles were excluded for the following reasons: lack of relevant outcome measures (n = 28), non-first-line therapy (n = 4), incompatible immunotherapy interventions (n = 4), inaccessible full texts (n = 8), unavailable or incomplete data (n = 7), ineligible study designs (n = 9), non-ICI immunotherapies such as vaccines or cell therapies (n = 1), inclusion of radiotherapy in the control arm (n = 1), and non-ICI-chemotherapy combination regimens (n = 1).

Following qualitative synthesis, eight eligible studies were ultimately selected for quantitative meta-analysis, comprising three randomized controlled trials (RCTs) and five non-randomized clinical trials (NRCTs). Notably, we included the Phase Ib/II study by Jia et al. (8), which evaluated a combination of fruquintinib (an anti-angiogenic agent), camrelizumab (an ICI), and chemotherapy. Although this regimen incorporates anti-angiogenic therapy, it was included as a specific subgroup due to the central role of ICI in the intervention and its high clinical relevance to the evolving landscape of first-line PDAC treatment.

### 3.2 Study characteristics and quality assessment

This systematic review identified eight clinical studies evaluating first-line immunotherapy for PDAC. Comprehensive details regarding study design, baseline patient characteristics, therapeutic regimens, and methodological quality are summarized in **Tables 1**, **2**, **Supplementary Appendix 1**, and **Supplementary Appendix 4**, respectively. The included randomized controlled trials (RCTs) comprised Renouf 2022 (9)(Phase II, Canada), Fu 2023 (10) (Phase II, China), and Jia 2025 (Phase Ib/II, China) (8). The five non-randomized clinical trials (NRCTs) included three Phase Ib/II trials (Weiss 2018, USA (11); Morizane 2024, Japan (12); Cheng 2024, China (13)) and two retrospective cohort studies (Song 2022 (14) and Chen 2023 (15) both from China). Geographically, China contributed the largest number of studies (n=5), followed by Canada, the United States, and Japan.

**Table 1:** Main characteristics of the 8 included studies for PDAC treatment.

Study ID	Design/phase	Pathology	Samples (N) [TG/CG]	TG median age (range)	CG median age	Liver met (%) [TG/CG]	Intervention scheme (experimental vs. control)
Renouf 2022 (9)	RCT/Phase II	mPDAC	119/61	64 (29–81)	65 (42-84)	NR	Gem+nab-P+Durvalumab+Tremelimumab vs. Gem+nab-P
Fu 2023 (10)	RCT/Phase II	m/rPDAC	55/55	61 (44–74)	62 (45–73)	80.0%/70.9%	Sintilimab + mFFX vs. mFFX
Jia 2025 (8)	RCT/Phase Ib/II	LA/mPDAC	45/45	59.0 (36.0–75.0)	59.0 (39.0–74.0)	51.1%/55.6%	NASCA (Fruquintinib + Camrelizumab + nab-P + S-1) vs. nab-P + Gem
Weiss 2018 (11)	NRCT/Phase Ib/II	mPDAC	17 (Single-arm)	56 (46–66)		NR	Pembrolizumab + Gem + nab-P
Song 2022 (14)	NRCT/Retrospective	Advanced PC	18 (Single-arm)	62 (38–80)		55.6%	PD-1 inhibitor + nab-P + Gem
Chen 2023 (15)	NRCT/Retrospective	LA/mPDAC	27 (Single-arm)	64 (46–77)		40.7%	PD-1 inhibitor + Gem + nab-P
Morizane 2024 (12)	NRCT/Phase 2	m/r PC	31 (Single-arm)	59.0 (39–75)		64.5%	Nivolumab + mFOLFIRINOX
Cheng 2024 (13)	NRCT/Phase Ib/II	LA/mPDAC	72 (Single-arm)	57 (43–71)		70.8%	Toripalimab + nab-P + Gem

**Table 2:** Best overall response and duration of response according to RECIST 1.1.

Study ID	Group	N	CR, n (%)	PR, n (%)	SD, n (%)	PD, n (%)	ORR, n (%)	DCR, n (%)	mDoR, mo (95% CI)
RCTs									
Renouf 2022 (9)	TG	119	0 (0%)	36 (30.3%)	45 (37.8%)	25 (21.0%)	36 (30.3%)	84 (70.6%)	NA
	CG	61	0 (0%)	14 (23.0%)	20 (32.8%)	15 (24.6%)	14 (23.0%)	35 (57.4%)	NA
Fu 2023 (10)	TG	55	0 (0%)	25 (45.5%)	19 (34.5%)	6 (10.9%)	25 (45.5%)	44 (80.0%)	6.8 (2.1–11.5)
	CG	55	0 (0%)	14 (25.5%)	26 (47.3%)	11 (20.0%)	14 (25.5%)	40 (72.7%)	6.4 (1.5–11.3)
Jia 2025 (8)	TG	45	0 (0%)	23 (51.1%)	18 (40.0%)	4 (8.9%)	23 (51.1%)	41 (91.1%)	8.1 (5.8–13.3)
	CG	45	0 (0%)	11 (24.4%)	29 (64.4%)	5 (11.1%)	11 (24.4%)	40 (88.9%)	6.0 (2.3–13.0)
NRCTs									
Weiss 2018 (11)	Single-arm group	12	0 (0%)	3 (25%)	8 (66.67%)	0 (0%)	3 (25%)	11 (91.67%)	NA
Song 2022 (14)	Single-arm group	18	1 (5.6%)	9 (50.0%)	5 (27.8%)	3 (16.7%)	10 (55.6%)	15 (83.4%)	NA
Chen 2023 (15)	Single-arm group	27	0 (0%)	10 (37.04%)	10 (37.04%)	7 (25.92%)	10 (37.04%)	20 (74.08%)	4.15 (IQR: 2.61–6.02)
Morizane 2024 (12)	Single-arm group	31	0 (0%)	10 (32.3%)	12 (38.7%)	7 (22.6%)	10 (32.3%)	22 (71.0%)	7.4 (3.5–21.9)
Cheng 2024 (13)	Single-arm group	72	1 (1.4%)	23 (31.9%)	41 (56.9%)	7 (9.7%)	24 (33.3%)	65 (90.3%)	6.2 (4.3–9.2)

The total sample size across the eight studies ranged from 17 to 180 patients. Renouf 2022 (9) represented the largest cohort (N = 180; experimental: 119, control: 61), while Weiss 2018 (11) was the smallest (N = 17, single-arm). Although the primary focus was metastatic PDAC (mPDAC), three studies (Jia 2025 (8), Chen 2023 (15), and Cheng 2024 (13)) also included patients with locally advanced (LA) disease, totaling 87 cases. Across the trial groups, the median age ranged from 56 years (Weiss 2018 (11)) to 64 years (Renouf 2022 (9) and Chen 2023 (15)). Liver metastasis prevalence varied significantly, with the highest proportion observed in Fu 2023 (10)(80.0%) and the lowest in Chen 2023 (15) (40.7%); notably, Renouf 2022 (9) and Weiss 2018 (11) did not provide specific data on metastatic sites. Performance status (ECOG PS) was well-distributed, with 151 patients scoring 0 and 310 scoring 1. Regarding tumor lateralization, the pancreatic body and tail accounted for 60.0%–77.8% of cases, while the pancreatic head accounted for 22.2%–50.0%.

All protocols utilized PD-1/PD-L1 inhibitors—including durvalumab, sintilimab, camrelizumab, pembrolizumab, nivolumab, or toripalimab—integrated with chemotherapy backbones. Six studies employed the Gem+nab-P regimen (Gemcitabine 1000 mg/m^2^ and nab-paclitaxel 125 mg/m^2^ on days 1 and 8 of a 21-day cycle), while two utilized mFOLFIRINOX/mFFX (14-day cycle). In the Jia 2025 (8) subgroup, the experimental arm received a triple combination of fruquintinib (200 mg QD PO), camrelizumab, and chemotherapy (nab-P + S-1–40 mg BID PO). Notable treatment discontinuations due to Grade≥3 non-hematologic toxicities were reported by Jia 2025 (8) (33.3%) and Song 2022 (14) (27.8%).

Methodological quality was rigorously evaluated. For RCTs, the Jadad scale (maximum 7 points) was applied; due to the open-label nature of these trials, blinding items were scored as 0. Fu 2023 (10) achieved 5 points (randomization +2, concealment +2, attrition +1), while Renouf 2022 (9) and Jia 2025 (8) each scored 4 points. All three RCTs were categorized as high-quality (score≥3). For NRCTs, the MINORS scale (maximum 16 points) was used. Morizane 2024 (12) and Cheng 2024 (13) achieved perfect scores (16/16). Weiss 2018 (11)scored 13 points, primarily due to limitations in patient representativeness. The retrospective studies (Song 2022 (14) and Chen 2023 (15)) each scored 11 points, reflecting deductions for lack of prospective data collection and pre-specified sample size calculations. The inclusion of 87 LA patients alongside the mPDAC cohort introduces potential biological heterogeneity, the implications of which are further addressed in the Discussion section.

### 3.3 Survival outcomes

#### 3.3.1 Clinical efficacy outcomes

The clinical efficacy parameters are summarized in **Table 3**, with median overall survival (mOS) and median progression-free survival (mPFS) serving as the primary evaluation endpoints.

**Table 3:** Summary of clinical efficacy outcomes (OS and PFS) in experimental vs. control groups.

Study ID	Group	N	mOS, mo (95% CI)	HR (95% CI)	mPFS, mo (95% CI)	HR (95% CI)
RCTs						
Renouf 2022 (9)	TG	119	9.8 (-)	0.94 (90% CI: 0.71–1.25)	5.5 (-)	0.98 (90% CI: 0.75–1.29)
	CG	61	8.8 (-)	Ref.	5.4 (-)	Ref.
Fu 2023 (10)	TG	55	10.9 (9.0–12.8)	1.07 (0.69–1.68)	5.9 (3.5–8.3)	0.93 (0.62–1.40)
	CG	55	10.8 (7.4–14.2)	Ref.	5.7 mo	Ref.
Jia 2025 (8)	TG	45	13.0 (10.5–16.1)	0.77 (0.47–1.28)	7.9 (5.9–9.4)	0.63 (0.40–0.99)
	CG	45	11.0 (8.4–15.3)	Ref.	5.3 (3.4–6.4)	Ref.
NRCTs						
Weiss 2018 (11)	TG	12	15.0 (12.0–15.0)	N/A	9.1 (4.9–15.3)	N/A
Song 2022 (14)	TG	18	NA	N/A	NA	N/A
Chen 2023 (15)	TG	27	16.4 (14.211–18.589)	N/A	6.4 (3.981–8.752)	N/A
Morizane 2024 (12)	TG	31	13.4 (10.6–16.6)	N/A	7.4 (3.9–9.2)	N/A
Cheng 2024 (13)	TG	72	8.9 (7.3–11.0)	N/A	5.6 (4.9–6.8)	N/A

In the three included randomized controlled trials (RCTs), survival data were as follows:

Renouf 2022 (N = 180) (9): The experimental group (n=119) achieved an mOS of 9.8 months compared to 8.8 months in the control group (n=61), with a hazard ratio (HR) of 0.94 (90% CI: 0.71–1.25). The mPFS was nearly identical between the two arms (5.5 months vs. 5.4 months; HR: 0.98; 90% CI: 0.75–1.29).

Fu 2023 (10)(N = 110): In the ITT population, the experimental group achieved an mOS of 10.9 months (95% CI: 9.0–12.8) compared with 10.8 months (95% CI: 7.4–14.2) in the control group (HR: 1.07; 95% CI: 0.69–1.68). The mPFS was 5.9 months (95% CI: 3.5–8.3) versus 5.7 months, respectively (HR: 0.93; 95% CI: 0.62–1.40), indicating no statistically significant survival advantage.

Jia 2025 (N = 104) (8): The experimental group reported an mOS of 13.0 months (95% CI: 10.5–16.1) vs. 11.0 months (95% CI: 8.4–15.3) in the control group (HR: 0.77; 95% CI: 0.47–1.28). The mPFS was 7.9 months in the experimental arm and 5.3 months in the control arm (HR: 0.63; 95% CI: 0.40–0.99).

Among the five NRCTs, median OS ranged from 8.9 months (Cheng 2024 (13)) to 16.4 months (Chen 2023 (15), 95% CI: 14.2–18.6). Median PFS values were 9.1 months (Weiss 2018 (11), 4.9–15.3), 7.4 months (Morizane 2024 (12), 3.9–9.2), 6.4 months (Chen 2023 (15), 3.98–8.75), and 5.6 months (Cheng 2024 (13), 4.9–6.8), and NR for Song 2022 (14). These NRCT data were summarized descriptively and were not included in comparative HR pooling.

#### 3.3.2 Survival evidence map analysis

**Figure 1:**
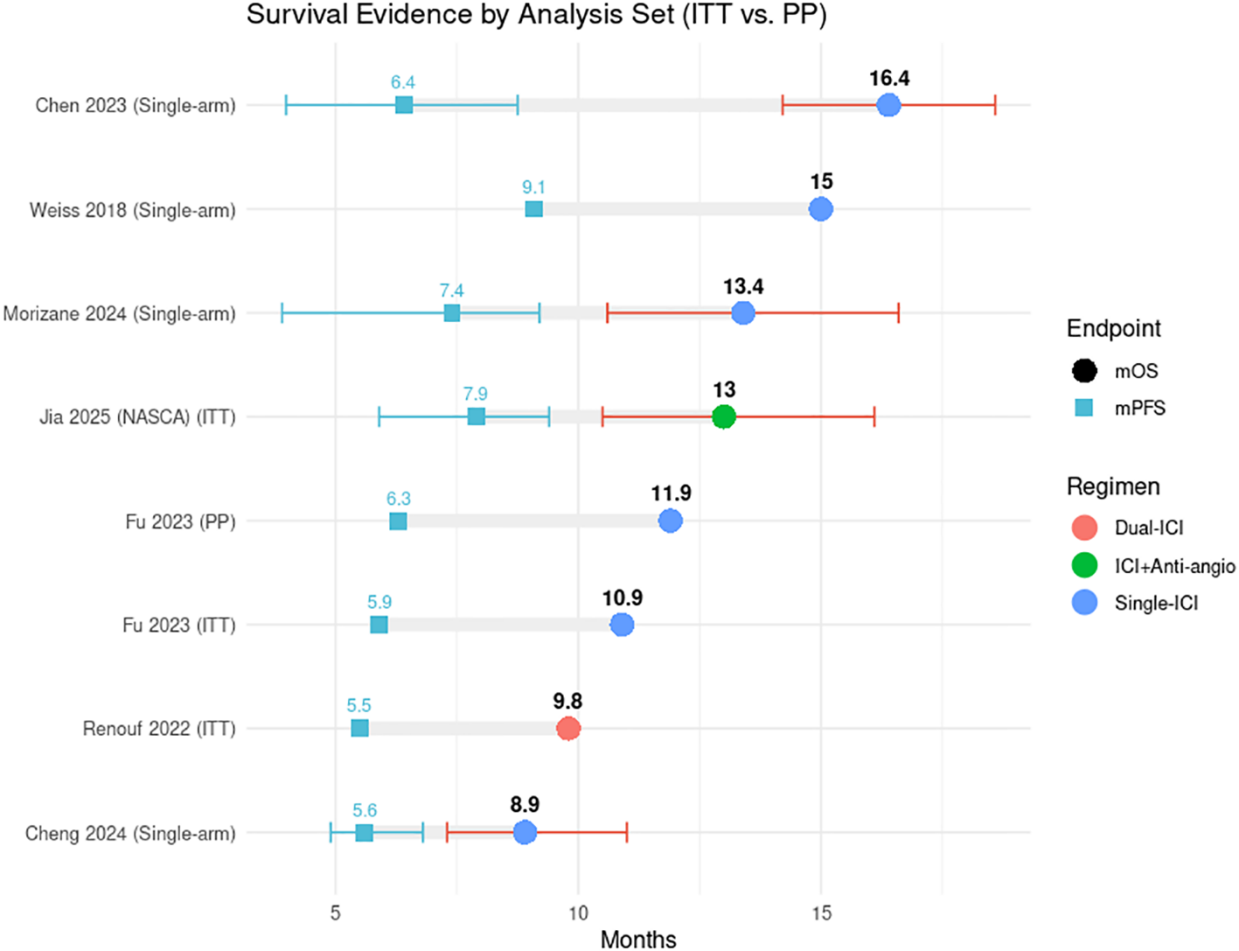
Comparative efficacy of ICI-based regimens in advanced PDAC A cumulative meta-analysis and subgroup evaluation of ITT vs. PP populations.

The survival evidence map (**Figure 1**) illustrates the distribution of median progression-free survival (mPFS) and median overall survival (mOS), alongside their corresponding 95% confidence intervals (CIs) across the included studies. The mOS ranged from 8.9 months (Cheng 2024 (13)) to 16.4 months (Chen 2023 (15), 95% CI: 14.2–18.6). NRCTs with mOS >13 months included Song 2022 (14) (NR), Weiss 2018 (11) (12.0–15.0), and Morizane 2024 (12) (10.6–16.6). Cheng 2024 (13) (7.3–11.0) and Renouf 2022 (9) (9.8 months) reported lower values. NRCT data are presented separately from RCT-only comparative analyses. Because **Figure 1** includes both RCT and NRCT studies, it is presented as a descriptive evidence map and was not used for comparative effect-size estimation.

The reported mPFS ranged from 5.6 months (Cheng 2024 (13), 4.9–6.8) to 9.1 months (Weiss 2018 (11), 4.9–15.3). In the RCT triple-therapy study Jia 2025 (8), mPFS was 7.9 months (5.9–9.4). NRCT data are presented separately for descriptive purposes.

When stratified by therapeutic strategy, the included studies were categorized into three regimen groups:

Single-ICI combinations (n=6 studies).
ICI plus anti-angiogenic therapy (Jia 2025 (8)): mOS 13.0 months; mPFS 7.9 months.
Dual-ICI combinations (Renouf 2022 (9); Durvalumab + Tremelimumab): mOS 9.8 months; mPFS 5.5 months.

#### 3.3.3 Meta-analysis of survival outcomes

**Figure 2:**
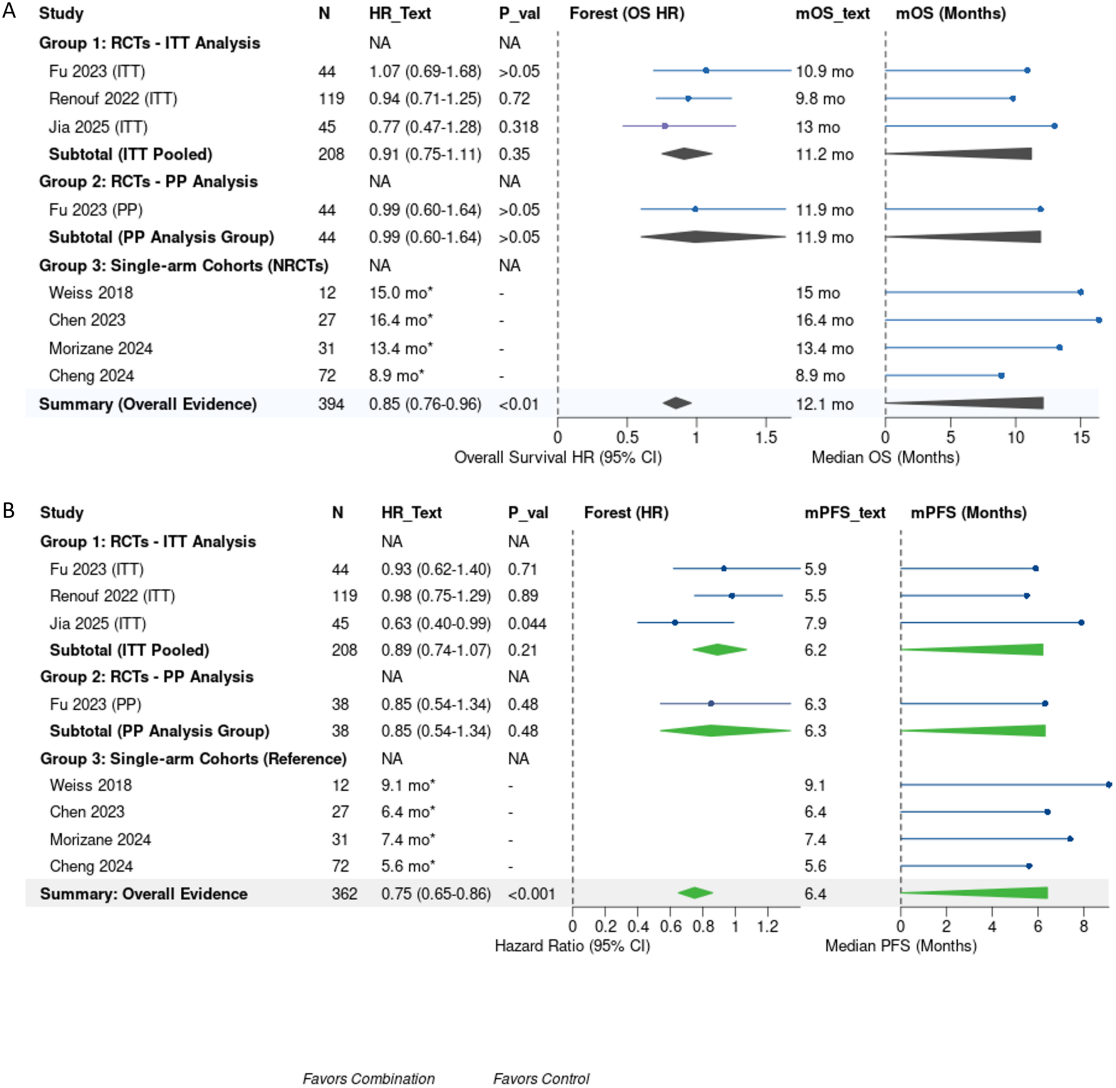
Integrated forest plots of survival trends for ICI-based combination therapies in advanced PDAC, featuring simplified pooling of hazard ratios (HRs) and median overall survival (OS) **(A)** and progression-free survival (PFS) **(B)**.

**Figure 3:**
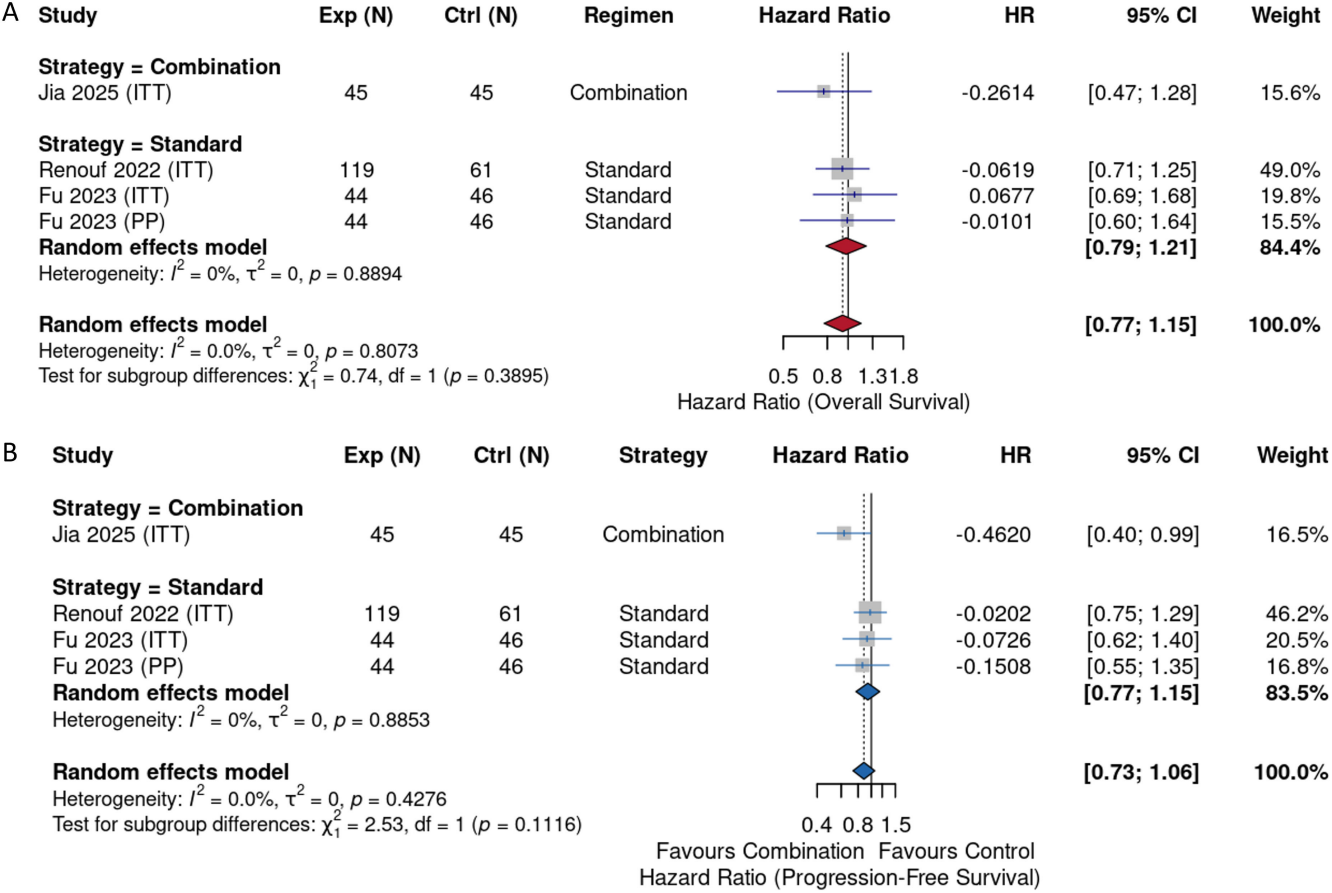
Forest plots for overall survival [OS, **(A)**] and progression-free survival [PFS, **(A)**] in RCTs of first-line ICI-based combination therapies.

In the RCT-only survival meta-analysis, ICI-based combinations showed favorable numerical trends in OS and PFS, but statistical significance was not reached for either pooled estimate. For OS, the pooled HR was 0.83 (95% CI: 0.65-1.06). For PFS, the pooled HR was 0.75 (95% CI: 0.53-1.05). NRCT and single-arm cohorts were summarized separately as descriptive survival data and were not included in comparative HR pooling (**Figures 2**, **3**).

#### 3.3.4 Subgroup meta-analysis of randomized controlled trials

Exploratory subgroup analysis of RCTs was performed based on therapeutic strategy to examine whether numerical differences in survival outcomes were consistent across regimen types. In the overall survival (OS) analysis, the triple-therapy arm (ICI + anti-angiogenic therapy; Jia 2025 (8), n = 90) demonstrated a hazard ratio (HR) of 0.77 (95% CI: 0.47–1.28), accounting for 21.5% of the pooled weight. The standard ICI-chemotherapy subgroup—comprising Renouf 2022 (9) and Fu 2023 (10) (n = 174)—showed an HR of 0.94 (95% CI: 0.71–1.25, weight: 57.6%) and 0.63 (95% CI: 0.38–1.05, weight: 20.9%), respectively. The pooled HR for this subgroup was 0.82 (95% CI: 0.56–1.19), with moderate heterogeneity (I^2^ = 45%). Aggregating all RCTs yielded an overall HR of 0.83 (95% CI: 0.65–1.06; I^2^ = 0.0%), with no significant difference observed between subgroups (P_interaction_ = 0.86).

Notably, although the pooled HR in the RCT cohort numerically favored ICI-based combinations, the difference was not statistically significant because the 95% CI crossed 1.0. No significant interaction was observed across treatment-strategy subgroups (P for interaction > 0.05), indicating that the apparent HR differences may reflect random variation rather than true effect modification. Regarding progression-free survival (PFS), Jia 2025 (8) reported an HR of 0.63 (95% CI: 0.40-0.99; weight: 28.7%). Within the standard ICI-chemotherapy subgroup, Renouf 2022 (9) and Fu 2023 (10) reported HRs of 0.98 and 0.60, respectively, resulting in a pooled subgroup HR of 0.79 (95% CI: 0.49-1.28), with substantial heterogeneity (I^2^ = 69.9%). The overall pooled HR for PFS was 0.75 (95% CI: 0.53-1.05; I^2^ = 58.0%), and the inter-subgroup interaction P-value was 0.49. Overall, these RCT subgroup analyses suggested numerical differences across therapeutic strategies, but interaction tests were not statistically significant. Therefore, these findings should be interpreted as exploratory and should not be considered evidence that any specific regimen subgroup derives a definitive subgroup-specific survival effect.

### 3.4 Assessment of tumor response: ORR and DCR

#### 3.4.1 Clinical tumor response

The radiological response parameters, including objective response rate (ORR), disease control rate (DCR), and median duration of response (mDoR), are presented in **Table 2**. Across these RCTs, objective responses were predominantly partial responses, whereas no complete responses were observed. In Renouf 2022 (9), the trial group achieved an ORR of 30.3% and a DCR of 70.6%, compared to 23.0% and 57.4% in the control arm, respectively. Fu 2023 (10) reported an ORR of 40.0% in the ITT population of the experimental arm versus 20.0% in the control arm, while the corresponding DCR values were 67.3% and 60.0% Similarly, Jia 2025 (8) recorded an ORR of 51.1% and a DCR of 91.1%, compared with 24.4% and 88.9% in the control arm. Across these RCTs, most objective responses were partial responses, whereas complete responses remained rare. Regarding the sustainability of these responses, the experimental arms in Jia 2025 (8) and Fu 2023 (10) yielded an mDoR of 8.1 months and 6.8 months, respectively, both of which compared favorably to their corresponding control arms (6.0 months and 6.4 months).

The extended mDoR observed in the Jia 2025 (8) triple-therapy arm is noteworthy and may partly explain the favorable PFS estimate reported in that study. However, this observation should be interpreted cautiously because it was derived from a single trial and requires confirmation in larger randomized studies. Among the five non-randomized clinical trials (NRCTs), response outcomes were summarized descriptively because these studies lacked concurrent chemotherapy control arms. Complete responses were uncommon and were limited to single cases in Song 2022 (14) (5.6%) and Cheng 2024 (13) (1.4%). In terms of overall efficacy, Song 2022 (14) exhibited an ORR of 55.6% and a DCR of 83.4%, while Cheng 2024 (13) reported an ORR of 33.3% and a high DCR of 90.3%. Weiss 2018 (11) and Morizane 2024 (12) recorded ORRs of 25.0% and 32.3%, respectively. Regarding the duration of response in these cohorts, Morizane 2024 (12) reported an mDoR of 7.4 months (95% CI: 3.5–21.9), while Chen 2023 (15) and Cheng 2024 (13) showed durations of 4.15 months (IQR: 2.6–6.0) and 6.2 months, respectively.

#### 3.4.2 Stratified meta-analysis of ORR and DCR

**Figure 4:**
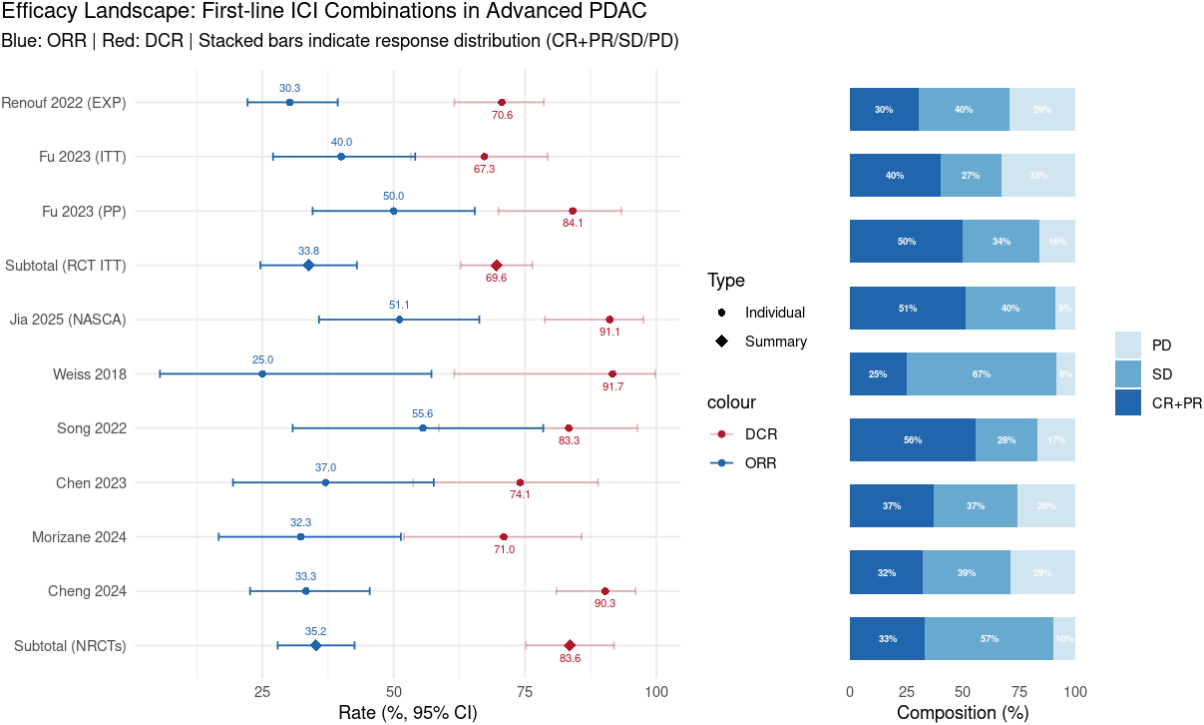
Response rates (ORR and DCR) of ICIs plus chemotherapy in PDAC.

**Figure 4** is presented as a descriptive visualization of ORR, DCR, and response composition across included studies. Because it includes different study designs and analysis populations, it was not used for formal comparative effect-size estimation. Therefore, the values shown in **Figure 4** were used to illustrate the distribution and general trend of tumor response across different study designs and analysis populations.

In the RCT ITT subgroup, the summary ORR was 33.8%, and the summary DCR was 69.6%. Jia 2025 (8), representing the ICI plus chemotherapy plus anti-angiogenic regimen, showed an ORR of 51.1% and a DCR of 91.1%. In the NRCT subgroup, the summary ORR was 35.2%, and the summary DCR was 83.6%. Among individual studies, Song 2022 (14) showed the highest ORR at 55.6%, followed by Jia 2025 (8) at 51.1%, Fu 2023 (10) per-protocol population at 50.0%, Fu 2023 (10) ITT population at 40.0%, Chen 2023 (15) at 37.0%, Cheng 2024 (13) at 33.3%, Morizane 2024 (12) at 32.3%, Renouf 2022 (9) at 30.3%, and Weiss 2018 (11) at 25.0%. For DCR, the highest values were observed in Weiss 2018 (11) at 91.7%, Jia 2025 (8) at 91.1%, Cheng 2024 (13) at 90.3%, Fu 2023 (10) per-protocol population at 84.1%, and Song 2022 (14) at 83.4%.

Stacked bar charts further delineate the proportions of partial response (PR), stable disease (SD), and progressive disease (PD) across individual studies. It should be noted that **Figure 4** is presented for descriptive purposes only, integrating different study designs and including both intention-to-treat (ITT) and per-protocol (PP) response patterns for Fu 2023 (10), and thus should be interpreted as an exploratory visualization rather than evidence of comparative treatment effect. In the Renouf 2022 (9) experimental arm, PR was observed in 36 cases (30.3%), SD in 45 cases (37.8%), and PD in 25 cases (21.0%). Similarly, Morizane 2024 (12) reported PR in 10 cases (32.3%), SD in 12 cases (38.7%), and PD in 7 cases (22.6%). Notably, Cheng 2024 (13) exhibited a high degree of disease stabilization, with 23 cases of PR (31.9%) and 41 cases of SD (56.9%), while only 7 cases (9.7%) experienced PD. The relatively low PD rate observed in the Cheng 2024 (13) and Jia 2025 (8) cohorts should be interpreted descriptively, given differences in study design, treatment regimen, and analysis population.

#### 3.4.3 Subgroup meta-analysis of ORR and DCR in RCTs

**Figure 5:**
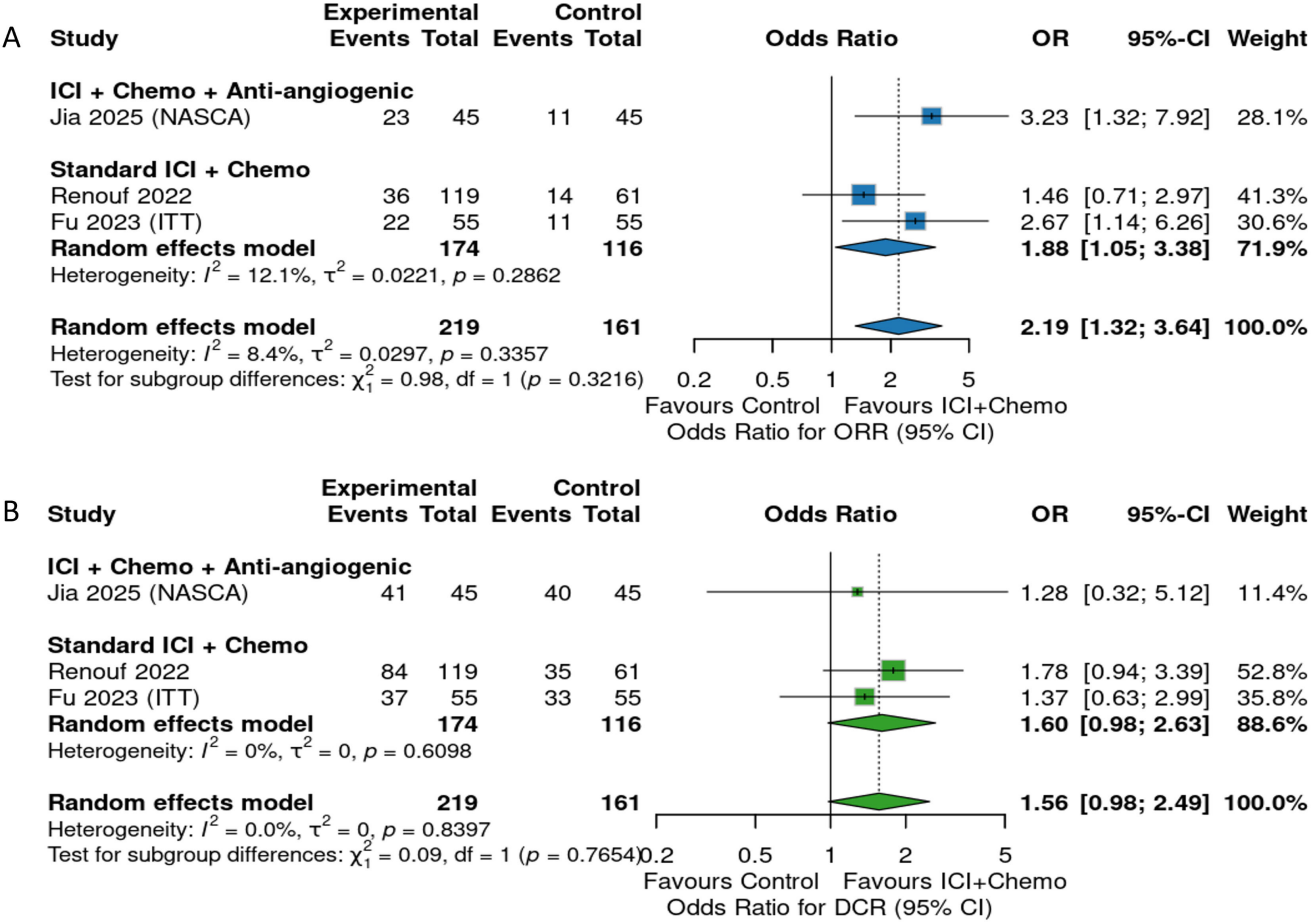
Forest plots of objective response rate ORR **(A)** and disease control rate DCR **(B)** in RCTs of first-line ICI-based combination therapies.

The formal comparative meta-analysis of tumor response was restricted to RCT ITT populations and was evaluated using odds ratios (ORs), as shown in **Figure 5**. In the objective response rate (ORR) subgroup analysis, the triple-therapy arm (ICI + anti-angiogenesis; Jia 2025 (8)) reported 23 responses among 45 patients in the experimental arm and 11 among 45 patients in the control arm, corresponding to an OR of 3.23 (95% CI: 1.32-7.92). Within the standard ICI-chemotherapy subgroup, Renouf 2022 (9) reported an OR of 1.46 (95% CI: 0.71-2.97), and Fu 2023 (10) reported an OR of 2.67 (95% CI: 1.14–6.26). The pooled OR for the standard ICI plus chemotherapy subgroup was 1.88 (95% CI: 1.05–3.38; I^2^ = 12.1%; P = 0.2862). Aggregating all RCTs, the pooled OR for ORR was 2.19 (95% CI: 1.32–3.64; I^2^ = 8.4%; P = 0.3357), indicating a statistically significant improvement in ORR with ICI-based combinations. No significant subgroup difference was observed between therapeutic strategies (P_interaction_ = 0.3216).

In the disease control rate (DCR) subgroup analysis, the Jia 2025 (8) experimental arm achieved disease control in 41 of 45 cases compared with 40 of 45 cases in the control arm (OR: 1.28; 95% CI: 0.32-5.12; weight: 20.6%). The standard ICI-chemotherapy subgroup—comprising Renouf 2022 (9) (OR: 1.78; 95% CI: 0.94–3.39) and Fu 2023 (10) (OR: 1.37; 95% CI: 0.63–2.99)—showed a pooled OR of 1.60 (95% CI: 0.98–2.63; I^2^ = 0%; P = 0.6098). The aggregate combined OR for DCR was 1.56 (95% CI: 0.98–2.49; I^2^ = 0%; P = 0.8397), with an inter-subgroup difference P-value of 0.7654. The observed OR values for DCR were numerically higher in the Jia 2025 (8) subgroup, although the between-subgroup difference was not statistically significant (P_interaction_ = 0.7654). Overall, DCR estimates were numerically higher in some ICI-based subgroups, but the pooled DCR result did not reach statistical significance and should be interpreted cautiously.

### 3.5 Subgroup analysis by clinical characteristics and treatment regimens

**Figure 6:**
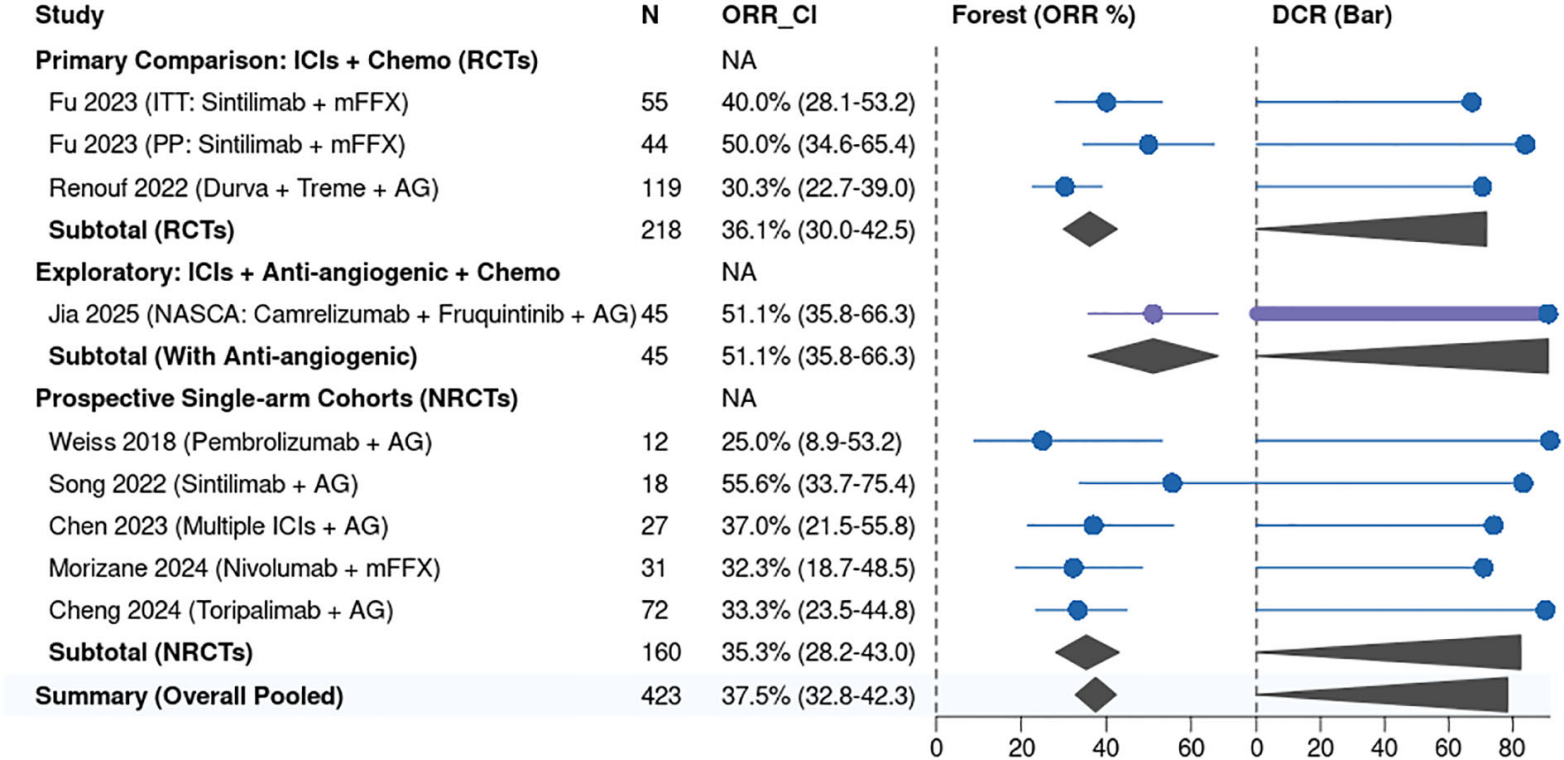
Integrated forest plots of clinical response trends for first-line ICI-based therapies in advanced PDAC, featuring simplified pooling of objective response rate (ORR) and disease control rate (DCR).

Exploratory stratified analyses of OS and PFS HRs were conducted according to chemotherapy backbone, use of anti-angiogenic therapy, median age, and liver metastasis burden. **Figure 6** summarizes subgroup-level distributions across study characteristics and treatment regimens for descriptive purposes. These analyses were intended to describe numerical patterns across clinically relevant strata rather than to establish definitive subgroup effects. Because the number of included studies was small and interaction P-values were >0.05 across subgroup analyses, these findings should be interpreted as exploratory observations rather than evidence of definitive subgroup-specific treatment effects.

### 3.6 Safety profile and Grade≥3 adverse events

**Figure 7:**
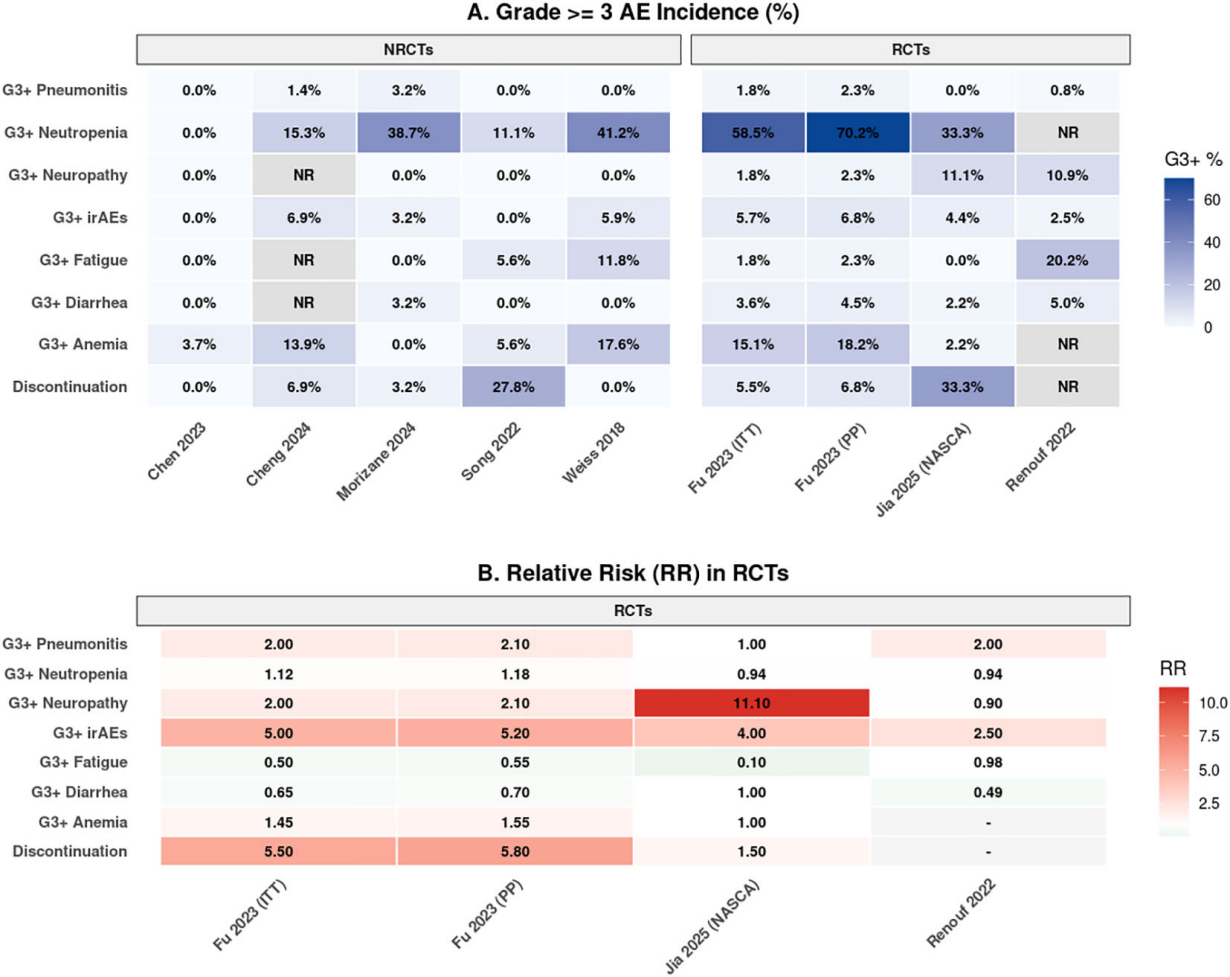
Heatmap visualization of safety profiles and relative risks (RR) for first-line ICI-based therapies in advanced PDAC, featuring simplified synthesis of Grade ≥3 adverse event incidence **(A)** and comparative relative risks within randomized trials **(B)**.

Grade ≥3 adverse events are summarized in **Table 4** and **Supplementary Table 5**, with heat-map and RCT relative-risk analyses presented in **Figure 7**.

**Table 4:** Incidences of Grade 3 or higher treatment-emergent and immune-related adverse events.

Study ID	Group	N (Safety)	Hematologic, n (%)			Non-hematologic, n (%)			irAEs, n (%)			
			Neutropenia	Anemia	Thrombocytopenia	Fatigue	Vomiting	Neuropathy	Any Grade	Grade ≥ 3	Pneumonitis	Hepatitis
RCTs												
Renouf 2022 (9)	TG	119	7 (6%)	0 (0%)	0 (0%)	24 (20.2%)	7 (5.9%)	13 (10.9%)	0 (0%)	3 (2.52%)	1 (0.8%)	0 (0%)
	CG	58	4 (7%)	0 (0%)	0 (0%)	2 (3.4%)	2 (3.4%)	7 (12.1%)	0 (0%)	0 (0%)	0 (0%)	0 (0%)
Fu 2023 (10)	TG	55	24 (43.6%)	4 (7.3%)	2 (3.6%)	1 (1.8%)	3 (5.5%)	1 (1.8%)	13 (23.6%)	3 (5.7%)	1 (1.8%)	1 (1.8%)
	CG	55	22 (40.0%)	3 (5.5%)	2 (3.6%)	2 (3.6%)	3 (5.5%)	0 (0%)	0 (0%)	0 (0%)	0 (0%)	0 (0%)
Jia 2025 (8)	TG	45	15 (33.3%)	1 (2.2%)	1 (2.2%)	0 (0%)	0 (0%)	5 (11.1%)	10 (22.2%)	2 (4.4%)	0 (0%)	0 (0%)
	CG	45	16 (35.6%)	1 (2.2%)	17 (37.8%)	2 (4.4%)	0 (0%)	0 (0%)	0 (0%)	0 (0%)	0 (0%)	0 (0%)
NRCTs												
Weiss 2018 (11)	Single-arm group	17	7 (41.18%)	3 (17.6%)	1 (5.9%)	2 (11.8%)	0 (0%)	0 (0%)	4 (23.5%)	1 (5.9%)	0 (0%)	1 (5.9%)
Song 2022 (14)	Single-arm group	18	2 (11.1%)	1 (5.6%)	1 (5.6%)	1 (5.6%)	0 (0%)	0 (0%)	5 (27.8%)	0 (0%)	0 (0%)	0 (0%)
Chen 2023 (15)	Single-arm group	27	0 (0%)	1 (3.7%)	0 (0%)	0 (0%)	0 (0%)	0 (0%)	0 (0%)	0 (0%)	0 (0%)	0 (0%)
Morizane 2024	Single-arm group	31	12 (38.7%)	0 (0%)	0 (0%)	0 (0%)	0 (0%)	0 (0%)	15 (48.4%)	1 (3.2%)	1 (3.2%)	0 (0%)
Cheng 2024 (13)	Single-arm group	72	11 (15.3%)	10 (13.9%)	NA	NA	NA	NA	NA	5 (6.9%)	1 (1.4%)	1 (1.4%)

#### 3.6.1 Safety profile in randomized controlled trials

Across the three RCTs, hematologic toxicities were the most frequently reported Grade ≥3 adverse events. Grade ≥3 neutropenia occurred at comparable rates between experimental and control arms in Fu 2023 (10) and Jia 2025 (8).Non-hematologic toxicities, including fatigue, peripheral neuropathy, and gastrointestinal events, varied across studies. Grade ≥3 irAEs were uncommon in the experimental arms and were not reported in control arms. Detailed event-specific rates are provided in **Table 4** and **Supplementary Table 5**.

#### 3.6.2 Safety profile in non-randomized clinical trials

In the five NRCTs, adverse events were summarized descriptively because these studies lacked concurrent chemotherapy control arms. Grade ≥3 hematologic toxicities, fatigue, pneumonitis, and hepatitis varied across cohorts, and irAE reporting was heterogeneous. Detailed safety data from NRCTs are presented in **Table 4** and **Supplementary Table 5**.

#### 3.6.3 Dose adjustments and discontinuation

Treatment discontinuation due to adverse events varied across studies, with higher discontinuation rates reported in some intensified regimens. Treatment-related mortality was uncommon and was reported only in Renouf 2022 (9). RCT relative-risk analyses for Grade ≥3 adverse events are presented in **Figure 7**. These analyses were based on available safety populations and should be interpreted in the context of limited event numbers and wide confidence intervals.

### 3.7 Pooled meta-analysis of various adverse reactions (Grade≥3) in RCTs

**Figure 8:**
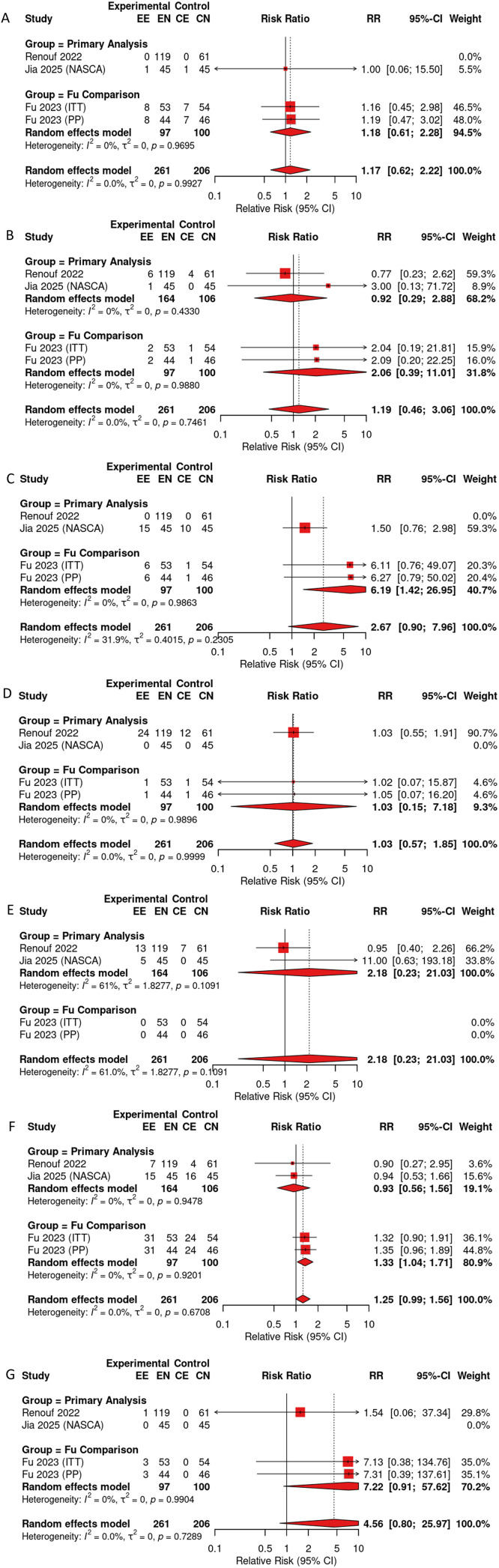
Forest plots for Grade≥ 3 adverse events, including Anemia **(A)**, Diarrhea **(B)**, discontinuation due to adverse events **(C)**, fatigue **(D)**, neuropathy **(E)**, neutropenia **(F)**, and pneumonitis **(G)** in RCTs of first-line ICI-based combination therapies.

The pooled relative risks (RRs) for Grade ≥3 adverse events across the three RCTs are presented in **Figure 8**. Overall, RCT-only pooled analyses did not show clear increases in Grade ≥3 anemia, diarrhea, fatigue, or neutropenia with ICI-based combinations compared with chemotherapy control arms.

For treatment discontinuation due to adverse events, the pooled RR was 1.62 (95% CI: 0.83–3.17; I^2^ = 0%), with no statistically significant difference between standard ICI-chemotherapy and triple-therapy subgroups. For Grade ≥3 neuropathy, the pooled RR was 1.91 (95% CI: 0.40–9.03; I^2^ = 33.9%), and the wide confidence interval indicates uncertainty due to limited event numbers. For Grade ≥3 pneumonitis, the pooled RR was 2.10 (95% CI: 0.22–19.93; I^2^ = 0%), also with a wide confidence interval reflecting rare events.

Detailed event-specific estimates, subgroup weights, and study-level RRs are provided in **Figure 8** and **Supplementary Table 5**.

#### 3.8 Sensitivity analysis and publication bias

**Figure 9:**
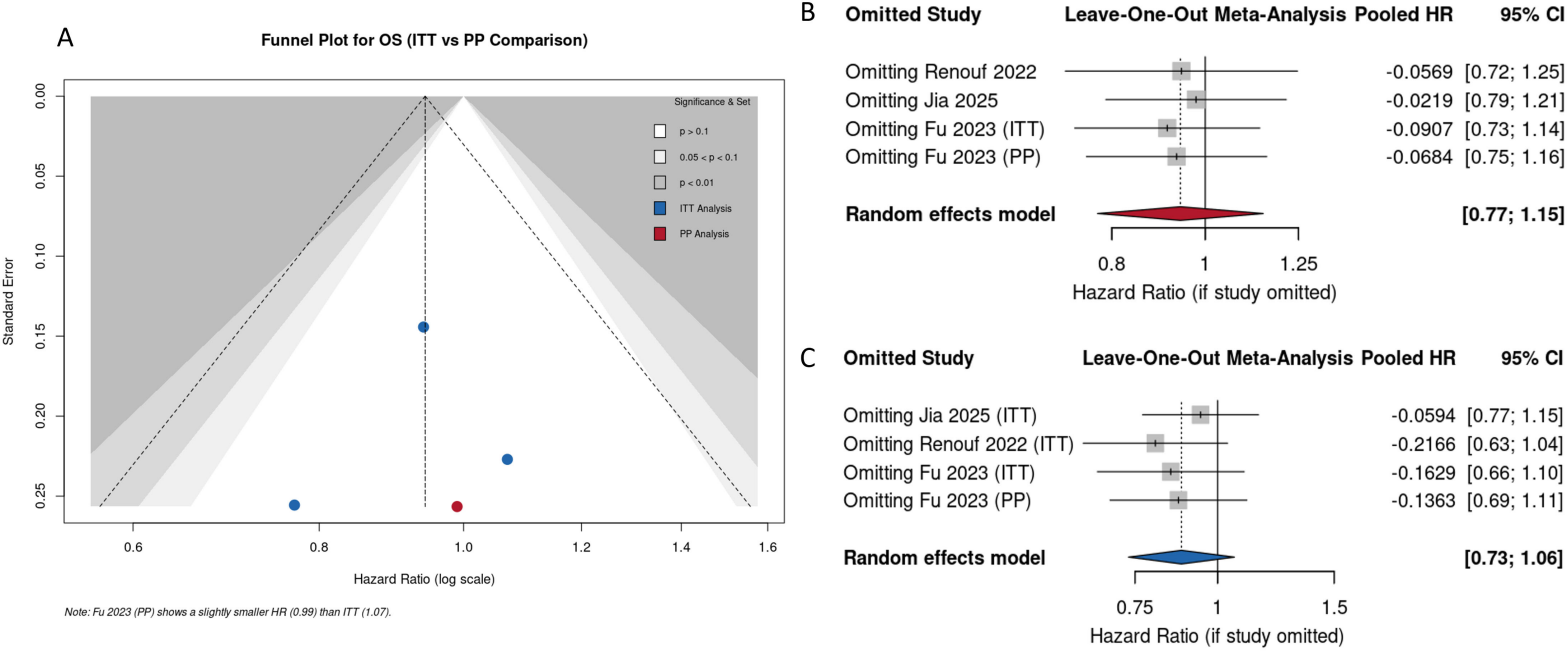
Publication bias and leave-one-out sensitivity analyses for overall survival (OS) and progression-free survival (PFS) in randomized controlled trials of first-line ICI-based combination therapies for advanced PDAC, including funnel plot for ITT vs PP comparison **(A)**, leave-one-out meta-analysis for OS **(B)**, and leave-one-out meta-analysis for PFS **(C)**.

Exploratory publication-bias assessment and leave-one-out influence diagnostics for OS and PFS are presented in **Figure 9**. These analyses were conducted to assess potential small-study effects and the influence of individual studies on pooled estimates, rather than to establish alternative statistically favorable conclusions. Detailed funnel plots for the primary endpoints, stepwise exclusion sensitivity analyses, and cumulative meta-analysis results are further provided in **Supplementary Figures 2**, **3** and **5**, respectively.

#### 3.8.1 Publication bias assessment

Publication bias was explored using funnel plots and contour-enhanced funnel plots for the primary endpoints. No clear pattern of small-study asymmetry was observed. However, because the number of included studies was small, especially in RCT-only analyses, funnel-plot interpretation and Egger’s/Begg’s tests had limited statistical power. Therefore, no firm conclusion regarding the presence or absence of publication bias can be made.

#### 3.8.2 Sensitivity analysis

Leave-one-out sensitivity analyses were performed to evaluate the influence of individual studies on the pooled estimates (**Supplementary Figure 3**). For OS, pooled HRs remained below 1.0 after sequential exclusion of individual studies, although the corresponding 95% confidence intervals generally crossed the null value. For ORR, pooled estimates varied across exclusion scenarios, ranging from OR = 1.79 (95% CI: 0.95–3.36) to OR = 2.50 (95% CI: 1.45–4.31). Overall, no single study substantially changed the direction of the pooled effects.

#### 3.8.3 Cumulative meta-analysis

Cumulative meta-analysis results are presented in **Supplementary Figure 5**. The pooled ORR increased from 25.0% after inclusion of the earliest study to 38.0% (95% CI: 31.8%–44.6%) after incorporation of all eligible studies. Similarly, the pooled mOS increased from 9.8 months to 12.0 months (95% CI: 9.7–14.2) as additional studies were added.

## 4 Discussion

### 4.1 Summary of efficacy in first-line treatment

This meta-analysis systematically reviewed and meta-analyzed the clinical efficacy of first-line immune checkpoint inhibitor (ICI) combination regimens in patients with advanced pancreatic ductal adenocarcinoma (PDAC) (16). By integrating data from eight clinical studies—including three randomized controlled trials (RCTs) and five non-randomized trials—comprising a total of 379 patients, we analyzed progression-free survival (PFS), overall survival (OS), objective response rate (ORR), and disease control rate (DCR) using pooled hazard ratios and odds ratios to assess the efficacy and safety of ICI-based combinations as first-line treatment for advanced PDAC.

#### 4.1.1 Overall survival outcomes

Regarding survival outcomes, the RCT-only pooled estimate numerically favored ICI-based combinations for OS (HR = 0.83, 95% CI: 0.65-1.06), but statistical significance was not reached. Therefore, the current randomized evidence suggests a favorable numerical survival trend rather than a definitive OS benefit. Descriptive survival outcomes from NRCTs appeared encouraging in some cohorts, but these studies lacked concurrent chemotherapy control arms and were not included in comparative HR pooling. Within the RCTs, Jia 2025 (8) reported an mOS of 13.0 months for the NASCA regimen (ICI plus anti-angiogenic therapy and chemotherapy), which was the longest median OS among the included randomized studies; however, this finding requires confirmation in larger trials.

The discrepancy between the descriptive survival observations and the RCT-only results may partly stem from the inherent “cold tumor” characteristics of PDAC. The PDAC tumor microenvironment (TME) is a highly immunosuppressive network, characterized by low effector immune cell infiltration and high densities of suppressive populations, including tumor-associated macrophages (TAMs), myeloid-derived suppressor cells (MDSCs), and regulatory T cells (Tregs) (17, 18). This network facilitates immune evasion through diverse mechanisms, such as secretion of pro-inflammatory cytokines, upregulation of immune checkpoint ligands, and intense metabolic competition (17, 19).

Furthermore, the hallmark dense fibrous stroma—which can constitute up to 80% of the tumor volume—functions as a physical and physiological barrier, leading to compressed vasculature and hypoxia. This impedes CD8+ T cell infiltration and limits delivery of therapeutic agents (18, 20). Key molecular drivers, such as fibroblast activation protein (FAP)-positive cancer-associated fibroblasts (CAFs), promote metastasis and chemo-immunotherapy resistance (21). Additional pathways, including WNT beta-catenin signaling, modulate TCF7 expression in CD4+ T cells to restrict CD8+ T cell recruitment, while CA9-mediated extracellular acidification impairs T cell effector function (22).

Our findings suggest a favorable but statistically non-definitive survival trend. Overcoming these multifaceted stromal and metabolic barriers—potentially via vascular normalization strategies such as the NASCA regimen—may be important for improving outcomes, but larger randomized trials are required to determine whether these numerical trends translate into definitive survival benefits.

#### 4.1.2 Progression-free survival improvements

Consistent with the numerical OS trend, the pooled HR for PFS was 0.75 (95% CI: 0.53–1.05), indicating a favorable but statistically non-definitive trend toward improved PFS.

In exploratory subgroup analysis, FOLFIRINOX/mFFX-based regimens showed favorable numerical PFS estimates (HR = 0.60). However, because the number of studies in each subgroup was small and interaction tests were not statistically significant, this observation should be interpreted cautiously and should not be taken as evidence of a definitive chemotherapy-backbone-specific benefit. This observation may have biological plausibility, as previous studies have shown that FOLFIRINOX is an active chemotherapy backbone in PDAC. However, the present subgroup analysis was underpowered and cannot determine whether FOLFIRINOX modifies the efficacy of ICI-based combinations.

A potential interaction between FOLFIRINOX and ICIs may be partly related to the pharmacodynamic properties of its components. Oxaliplatin and irinotecan have been reported to induce immunogenic cell death (ICD) (23). By promoting the release of damage-associated molecular patterns, these cytotoxic agents may facilitate dendritic-cell maturation and antigen presentation (24). This mechanism may provide a possible rationale for combining FOLFIRINOX with ICIs. However, the current subgroup findings remain exploratory and hypothesis-generating, and larger randomized studies are required to determine whether chemotherapy backbone modifies the efficacy of ICI-based combinations.

#### 4.1.3 Tumor response assessment

Regarding radiological response, the pooled odds ratio (OR) for objective response rate (ORR) across RCTs was 2.19 (95% CI: 1.32-3.64), indicating a statistically significant improvement in ORR with ICI-based combinations. The NASCA regimen (Jia 2025 (8)), representing a triple-therapy combination of ICI, chemotherapy, and an anti-angiogenic agent, showed encouraging response estimates, with an ORR of 51.1% and a DCR of 91.1%. While PDAC has traditionally been resistant to immunotherapy due to its complex tumor microenvironment (TME) and inherent immunosuppressive barriers (18, 19), these results suggest that selected combination strategies may partially modulate the immunosuppressive tumor microenvironment, although this hypothesis requires prospective validation.

The potential mechanisms driving this improved tumor response are multifaceted. Beyond the induction of immunogenic cell death (ICD) by chemotherapy, cytotoxic agents facilitate the release of tumor-associated antigens (TAAs), thereby activating antigen-presenting cells (APCs) to sustain anti-tumor immunity (24). Furthermore, the addition of anti-angiogenic agents, such as fruquintinib, may represent one possible contributor to the observed response pattern. These agents contribute to the normalization of aberrant tumor vasculature, alleviating hypoxia and interstitial fluid pressure, which increases effector T-cell infiltration and reduces the prevalence of immunosuppressive populations (25, 26). This potential reshaping of the TME may address one hypothesized limitation of previous PDAC immunotherapy failures, where monotherapies—whether ICI or anti-angiogenic—were insufficient to penetrate the dense stroma and restore immune surveillance (27).

Overall, these findings support further investigation of tripartite strategies combining ICIs, anti-angiogenic therapy, and cytotoxic chemotherapy in first-line PDAC, but current evidence is insufficient to define these regimens as a new treatment paradigm.

### 4.2 Safety considerations and the benefit-risk balance

Following the systematic evaluation of efficacy, this section examines the safety profile and the critical balance between clinical benefit and treatment-related risk for ICI-chemotherapy combinations in first-line PDAC. The safety analysis incorporates data from eight clinical studies, including three randomized controlled trials (RCTs) and five non-randomized trials (NRCTs), and evaluates hematologic and non-hematologic toxicities, as well as immune-related adverse events (irAEs), treatment discontinuation, and treatment-related mortality.

Safety assessment is particularly crucial for malignancies like PDAC, which are characterized by a dismal prognosis and limited therapeutic windows. Achieving therapeutic breakthroughs while carefully managing toxicity is essential to preserve patient quality of life and ensure the sustainability of treatment.

#### 4.2.1 Overall safety profile

Our analysis of eight clinical studies, including three RCTs and five non-randomized trials (NRCTs) encompassing a total of 379 patients, reveals that hematologic toxicity remains the most prevalent adverse event associated with ICI-chemotherapy combinations. This profile largely mirrors that of standard-of-care chemotherapy backbones.

Specifically, across the three RCTs, the incidence of Grade≥3 neutropenia ranged from 33.3% to 43.6%. For example, Fu 2023 (10) reported a neutropenia rate of 43.6% in the experimental arm versus 40.0% in the control arm (RR: 1.09; 95% CI: 0.70–1.70), showing no significant escalation of myelosuppression with the addition of ICIs. Similarly, Jia 2025 (8) observed rates of 33.3% and 35.6%, respectively (RR: 0.94; 95% CI: 0.53–1.66). These findings align with historical benchmarks for FOLFIRINOX and GnP regimens, where Grade≥3 neutropenia typically ranges from 30% to 50%.

Notably, the risk of neutropenia—particularly in FOLFIRINOX-based protocols—is closely linked to UGT1A1 gene polymorphisms. Patients homozygous for UGT1A1*28 and UGT1A1*6 are at higher risk for severe hematologic toxicity (28), underscoring the need for genetic screening to guide dose individualization. While the incidence of Grade≥3 anemia remained low (pooled RR: 1.25; 95% CI: 0.35–4.51), Jia 2025 (8) reported a lower incidence of thrombocytopenia in the experimental group (2.2%) compared to the control group (37.8%), which may reflect sample size limitations or baseline cohort differences.

Regarding non-hematologic toxicities, fatigue was common. Renouf 2022 (9) reported similar fatigue rates (~20%) between groups, whereas the RR for Grade≥3 fatigue in Jia 2025 (8) was notably low (0.10), suggesting no clear increase in severe fatigue in the available RCT data, although this estimate should be interpreted cautiously because it was based on limited events.

Peripheral neuropathy remains a key concern for taxane- or oxaliplatin-containing regimens, with rates of approximately 11% in both Renouf 2022 (9) and Jia 2025 (8). Notably, the RR for Grade≥3 neuropathy in the Jia 2025 (8) triple-therapy arm reached 11.00. Although the wide 95% CI (0.63–193.18) encompasses the null line due to small event numbers, this trend suggests that combining fruquintinib and camrelizumab with chemotherapy may potentiate neurotoxicity. Prior studies have suggested a possible association between chemotherapy-induced peripheral neuropathy and treatment exposure or outcomes in selected chemotherapy settings (29); however, the current meta-analysis was not designed to evaluate CIPN as an efficacy biomarker. Finally, gastrointestinal toxicities such as vomiting remained infrequent (5.5%–5.9%) across RCTs, likely reflecting the effectiveness of modern prophylactic antiemetic protocols.

#### 4.2.2 Immune-related adverse events

Immune-related adverse events (irAEs) represent a distinct pharmacological concern, characterized by auto-inflammatory mechanisms fundamentally different from the off-target toxicities of conventional cytotoxic agents. In our meta-analysis, RCT-only data showed that no irAEs were documented in any of the control arms, whereas the experimental arms exhibited an incidence of 22.2% to 23.6%, with Grade≥3 events accounting for 4.4% to 5.7%. These findings align with a large-scale systematic review of 29 RCTs involving 8,576 patients, indicating that PD-1/PD-L1 inhibitors have a unique toxicity profile, with overall risk at any grade often lower than that of conventional chemotherapy or CTLA-4 inhibitors (30).

Among the various irAEs, immune-mediated pneumonitis is particularly concerning due to its potential severity. Our pooled analysis yielded a relative risk (RR) of 2.10 (95% CI: 0.22–19.93) for Grade≥3 pneumonitis. Although the wide confidence interval reflects the low event frequency, these events warrant careful monitoring. For example, Renouf 2022 (9) reported a 0.8% incidence of high-grade pneumonitis. The general incidence of ICI-related pneumonitis is approximately 2%–5%, and it remains a leading cause of treatment-related mortality. Existing meta-analytic data suggest that PD-1 inhibitors carry a significantly higher risk for pulmonary toxicity compared to other organ-specific toxicities (OR: 4.7), requiring early radiologic screening and prompt glucocorticoid intervention (30).

In the non-randomized cohorts, Morizane 2024 (12) reported the highest overall irAE incidence at 48.4%, though severe cases (Grade≥3) were rare (3.2%). Weiss 2018 (11) and Song 2022 (14) reported overall irAE rates of 5.9% and 27.8%, respectively, with Grade≥3 events absent in Song 2022 (14). Specific organ toxicities, such as high-grade hepatitis, occurred sporadically in Weiss 2018 (11) (5.9%) and Cheng 2024 (13) (1.4%). Collectively, these results, supported by real-world evidence from Chinese cohorts (31), suggest that ICI-based combinations introduce a distinct spectrum of toxicities; however, their safety profile appeared manageable under clinical trial conditions with careful patient selection and monitoring.

#### 4.2.3 Treatment discontinuation and mortality

Treatment discontinuation serves as a key metric for assessing the clinical tolerability and feasibility of combination regimens. In our meta-analysis, the pooled relative risk (RR) for treatment interruption due to adverse events, based on RCT-only data, was 1.62 (95% CI: 0.83–3.17). Although numerically elevated, the confidence interval encompassing the null line suggests that the addition of ICIs to chemotherapy does not statistically increase the risk of premature treatment termination across the broader study population.

However, a granular analysis reveals notable variations among specific protocols. In the Jia 2025 (8) study, the discontinuation rate due to non-hematologic toxicities was 33.3%, the highest among included trials, suggesting that intensified triple-therapy regimens (ICI + chemotherapy + anti-angiogenic agent) may impose significant physiological stress, potentially due to overlapping toxicities. Similarly, Song 2022 (14) reported a discontinuation rate of 27.8%, whereas Chen 2023 (15) and Weiss 2018 (11) observed no therapy-terminating adverse events (0%). This heterogeneity likely reflects differences in enrollment criteria, management experience, and baseline patient performance status.

Regarding treatment-related mortality, Renouf 2022 (9) reported a rate of 1.7% in the experimental arm, slightly lower than 3.4% in the control arm. Importantly, none of the remaining seven studies documented any treatment-related deaths. These low incidences suggest that ICI-chemotherapy combinations were feasible under stringent clinical trial protocols with careful patient selection and monitoring. However, the limited sample size and selected trial populations preclude firm conclusions about treatment-related mortality in broader clinical practice. Overall, complex multidrug regimens may carry a higher risk of treatment discontinuation and should be evaluated carefully in future trials.

### 4.3 Clinical heterogeneity and its implications

Low statistical heterogeneity does not imply clinical homogeneity. The included studies varied substantially in multiple aspects that may influence both efficacy and safety outcomes. These differences included the class of ICI used (PD-1 inhibitors, PD-L1 inhibitors, or CTLA-4 combinations), the chemotherapy backbone (Gemcitabine + nab-Paclitaxel versus modified FOLFIRINOX), and the inclusion of anti-angiogenic agents, which may modulate immune cell infiltration and the tumor microenvironment. Geographic and population differences were also present, with patients enrolled in China, Japan, Canada, and the United States, potentially introducing variability in genetics, clinical practice, and supportive care.

Disease stage varied across studies, with some including both locally advanced (LAPC) and metastatic PDAC, contributing to biological heterogeneity. Differences between populations analyzed for safety versus efficacy, as well as study design factors—such as single-arm studies prone to selection bias versus randomized controlled trials—further contributed to variability in outcomes. These clinical heterogeneities highlight that pooled results should be interpreted with caution.

While statistical measures such as I^2^ provide an estimate of variability, they do not fully capture differences in trial design, patient characteristics, or treatment regimens that can meaningfully impact clinical outcomes. Understanding these factors is essential for accurately contextualizing the efficacy and safety of ICI-based combination therapies in first-line PDAC management.

#### 4.3.1 Publication bias assessment and its implications

Publication-bias assessment was exploratory because of the small number of included studies. Although the funnel plots did not show a clear pattern of small-study asymmetry, the limited number of available trials reduced the reliability of funnel-plot interpretation and the statistical power of Egger’s and Begg’s tests. Therefore, these analyses should be interpreted as exploratory diagnostics rather than evidence confirming the absence of publication bias.

#### 4.3.2 Sensitivity analysis and result stability

Leave-one-out sensitivity analysis was performed to examine whether any individual study had a disproportionate influence on the pooled estimates. For OS, the pooled HRs remained numerically below 1.0 after sequential exclusion of individual studies, suggesting that the numerical direction of the estimate was not entirely driven by a single trial. However, because the confidence intervals generally remained wide and crossed the null line, these results should be interpreted as exploratory evidence of directional consistency rather than confirmatory evidence of statistical robustness or definitive survival benefit.

For objective response rate (ORR), excluding Fu 2023 (10) resulted in a pooled OR of 2.07 (95% CI: 0.95–4.49), with the confidence interval crossing the null line, although the point estimate remained above 1.0. This suggests that the statistical significance of the ORR estimate may be sensitive to the inclusion of individual RCTs, particularly in the context of a small number of trials. Excluding Jia 2025 (8) or Renouf 2022 (9) produced pooled ORs of 1.83 (95% CI: 1.07–3.12) and 2.50 (95% CI: 1.45–4.31), respectively, but these exclusion-based results should be interpreted as supplementary influence diagnostics.

Regarding OS, although HRs remained numerically below 1.0 across sequential exclusions, the 95% confidence intervals consistently encompassed the null line, reflecting limited statistical certainty. These findings suggest that the numerical direction of the OS estimate was not solely attributable to a single study; however, they do not establish a definitive survival benefit. Proper consideration of inter-study heterogeneity and cautious interpretation of sensitivity analyses are essential when synthesizing evidence in the complex context of PDAC immunotherapy (32).

#### 4.3.3 Cumulative meta-analysis and evidence evolution

Cumulative meta-analysis provides a temporal perspective on the development of clinical evidence, allowing for the evaluation of how successive trials influence overall estimates of treatment efficacy. This approach quantifies the current strength of evidence and highlights evolving trends that can inform clinical practice.

For objective response rate (ORR), the cumulative analysis showed a progressive increase over time. Beginning with Weiss 2018 (11) (25.0%), the pooled ORR gradually increased as additional studies were incorporated, eventually stabilizing at 38.0% (95% CI: 31.8–44.6%). This pattern may reflect changes in study design, patient selection, and combination strategies over time, but it should be interpreted cautiously because cumulative estimates were based on a small number of heterogeneous studies.

Similarly, the cumulative median overall survival (mOS) started at 9.8 months (Renouf 2022 (9)) and increased progressively to 12.0 months (95% CI: 9.7–14.2) following the inclusion of subsequent trials, including Fu 2023 (10), Chen 2023 (15), and Morizane 2024 (12). The accumulation of evidence for both ORR and mOS was not strictly monotonic, reflecting how new data continuously recalibrate the global estimate. This dynamic pattern underscores the importance of sequential evidence integration in validating novel therapeutic paradigms, consistent with observations in biomarker development research.

Overall, cumulative analyses provide a descriptive view of how the evidence has evolved over time, but they do not establish definitive survival or response benefits given the limited number of studies and the heterogeneity of included regimens.

#### 4.3.4 Clinical implications

Drawing on the assessments of heterogeneity and sensitivity analyses, this meta-analysis provides several implications for future research and cautious clinical interpretation. The cumulative meta-analysis suggests that pooled estimates for ORR and mOS have become more stable over time; however, the limited number of included studies and the small sample sizes of several trials restrict the certainty of these observations. Therefore, first-line ICI-chemotherapy combinations should still be regarded as investigational strategies with encouraging antitumor activity rather than established standard regimens.

Despite clinical heterogeneity across trials, the available evidence suggests improved tumor response and favorable numerical survival trends. However, definitive survival benefit has not been established in RCT-only analyses. Clinicians should therefore interpret these findings cautiously and consider individual patient characteristics, including performance status and molecular profiles, when evaluating treatment options. Subgroup findings related to age, liver metastasis burden, chemotherapy backbone, and anti-angiogenic therapy should not be used to guide definitive treatment selection at this stage, because these analyses were exploratory and underpowered.

It should be noted that the number of included studies remains limited (n=8), with several trials being early-phase or small-sample cohorts. This constrains statistical power and highlights the need for large, multicenter randomized controlled trials with extended follow-up to determine whether these numerical trends translate into durable survival benefits. In addition, standardized treatment protocols and rigorous patient stratification, particularly regarding molecular subtypes and PD-L1 expression, are needed to improve inter-study comparability and identify patient subgroups that may derive greater benefit from ICI-based therapy.

### 4.4 Strengths and limitations

Following a systematic evaluation of the efficacy and safety of ICI-chemotherapy combinations in first-line PDAC, this section provides an objective analysis of the study’s strengths and limitations. Such a balanced perspective is essential for the accurate interpretation of our findings and for guiding the clinical application of these results.

#### 4.4.1 Strengths

The primary advantages of this meta-analysis are demonstrated in several key dimensions. First, regarding methodological rigor, this study strictly adhered to PRISMA guidelines, incorporating 8 clinical trials with a total of 379 patients. While seemingly modest, this sample size is substantial within the specialized niche of PDAC immunotherapy—a field often hampered by limited trial volumes and prolonged follow-up requirements (33, 34). Second, this study provides a multidimensional clinical evaluation. Beyond the primary endpoint of OS, we conducted systematic analyses of PFS, ORR, and DCR, while simultaneously characterizing the unique safety spectrum of these combinations. This comprehensive approach helps summarize an emerging evidence base in a field where standard chemotherapy benefits remain limited (35).

Furthermore, this study incorporated sensitivity analyses, subgroup interaction tests, and publication-bias diagnostics to examine the consistency and limitations of the available evidence. Given the small number of included studies, these analyses should be interpreted as supportive and exploratory rather than definitive. The cumulative meta-analysis provides an additional temporal perspective on how evidence for ICI-based regimens has evolved.

#### 4.4.2 Limitations

Despite the strengths of this meta-analysis, several limitations warrant cautious interpretation of the findings. First, the overall sample size remains modest. Although 379 patients were included, this number is relatively small for achieving definitive statistical power, particularly for overall survival (OS). This limitation is common in pancreatic cancer research due to the disease’s aggressive nature and the scarcity of large-scale, high-quality trials (36, 37). Consequently, while survival trends appear favorable, the 95% confidence intervals encompassing the null line in some analyses indicate that these observations should be interpreted as preliminary. Additionally, the small number of included studies limits the reliability of publication-bias assessment; funnel plots and Egger’s or Begg’s tests were underpowered and cannot exclude potential publication bias.

Second, methodological and clinical heterogeneity is substantial. The included studies differed in ICI agents (PD-1 versus PD-L1 inhibitors), chemotherapy backbones, and the use of anti-angiogenic therapy. Variations in study design between randomized controlled trials (RCTs) and non-randomized clinical trials (NRCTs) further contribute to this heterogeneity. Such differences limit the ability to draw definitive comparative conclusions and reflect the lack of standardized treatment protocols and patient stratification across international centers (38, 39).

Third, the analysis relied on aggregated study-level data rather than individual patient data (IPD), which precludes in-depth subgroup analyses and limits the evaluation of patient-level effects based on molecular or metabolic characteristics. This approach is inherently less precise than IPD-based meta-analyses and may be susceptible to reporting biases.

Finally, variability in follow-up durations and potential selection bias in smaller cohorts may influence the comparability of survival outcomes. As additional high-quality data emerge from diverse populations, particularly from specialized cohorts in Japan and China, further trials will be essential to test and refine the observations presented herein (40).

Overall, these limitations underscore the need to interpret the efficacy and safety trends reported in this study conservatively. While the findings provide preliminary evidence supporting first-line ICI-chemotherapy combinations in advanced PDAC, definitive clinical recommendations require confirmation from larger, rigorously designed trials.

## 5 Conclusion

This meta-analysis systematically evaluated the clinical efficacy and safety of first-line immune checkpoint inhibitors (ICIs) in combination with chemotherapy for patients with advanced pancreatic ductal adenocarcinoma (PDAC). Adhering strictly to PRISMA guidelines, we synthesized evidence from eight clinical trials (3 RCTs and 5 NRCTs) involving 379 patients to delineate the survival benefits, radiological responses, and safety spectrum of these emerging regimens.

### 5.1 Efficacy and clinical activity

This meta-analysis suggests that first-line ICI-based combination regimens may provide encouraging antitumor activity in advanced PDAC, particularly in terms of radiological response. In RCT-only analyses, ORR was improved, whereas OS and PFS showed favorable but statistically non-definitive numerical trends because the confidence intervals crossed the null line. The anti-angiogenic-containing NASCA regimen yielded encouraging response and PFS estimates; however, these findings should be considered exploratory and require confirmation in larger randomized trials. Overall, the current evidence supports further investigation of first-line ICI-based combination strategies, but does not establish a definitive survival benefit. Larger, adequately powered randomized trials with standardized patient stratification and biomarker assessment are needed to determine whether these numerical trends translate into statistically robust and clinically meaningful survival improvements.

### 5.2 Safety and tolerability

In terms of safety, ICI-based combination regimens showed a toxicity profile broadly consistent with the known adverse events of cytotoxic chemotherapy backbones. Hematologic toxicity was the most common adverse event, with Grade ≥3 neutropenia occurring in 33.3%–43.6% of patients, consistent with historical benchmarks for standard chemotherapy. Immune-related adverse events (irAEs) were observed in 22.2%–48.4% of patients, whereas severe (Grade ≥3) irAEs were uncommon, occurring in 4.4%–5.7% of cases. Treatment-related mortality was rare and reported only in specific trials, suggesting that these combinations may be feasible under careful clinical monitoring, although safety conclusions remain limited by small sample sizes and selected trial populations.

## Data Availability

The data supporting the findings of this study are available from the corresponding author upon reasonable request. All included studies were obtained from published literature in PubMed, Embase, Cochrane Library, Web of Science, Scopus, CNKI, Wanfang Data, VIP, and CBM.

## Supplementary material

Supplementary Figure 1 PRISMA flow diagram illustrating the systematic identification, screening, eligibility, and inclusion process of randomized controlled trials for the meta-analysis.

Supplementary Figure 2 Publication bias and small-study effect analysis of clinical outcomes, including contour-enhanced funnel plots for assessment of publication bias (Panels A–D) in RCTs of first-line ICI-based combination therapies.

Supplementary Figure 3 Leave-one-out sensitivity analysis for the primary outcomes (ORR and OS) in RCTs of first-line ICI-based combination therapies.

Supplementary Figure 4 Heatmap of Grade 3 or higher adverse events (General/Chemo, Hematological, and Immune-related) across included RCTs of first-line ICI-based combination therapies.

Supplementary Figure 5 Cumulative meta-analysis of clinical outcomes over publication years in RCTs of first-line ICI-based combination therapies.

## Glossary

PDAC: Pancreatic ductal adenocarcinoma
mPDAC: Metastatic pancreatic ductal adenocarcinoma
RCT: Randomized controlled trial
NRCT: Non-randomized controlled trial
TG: Treatment group (Experimental group)
CG: Control group
N: Sample size
M/F: Male-to-female ratio
ECOG PS: Eastern Cooperative Oncology Group Performance Status
B&T: Body and tail (Tumor location)
NCT: ClinicalTrials.gov identifier
Ref.: Reference
DOI: Digital Object Identifier
ICI: Immune checkpoint inhibitor
Gem: Gemcitabine
nab-P: Albumin-bound paclitaxel (nab-paclitaxel)
S-1: Tegafur
mFFX/mFOLFIRINOX: Modified FOLFIRINOX (Fluorouracil, Leucovorin, Oxaliplatin, and Irinotecan)
Dur: Durvalumab
Tre: Tremelimumab
Sin: Sintilimab
Cam: Camrelizumab
Pem: Pembrolizumab
Nivo: Nivolumab
Tori: Toripalimab
Fru: Fruquintinib
IV: Intravenous
PO: Per os (Oral)
q14d/q21d/q28d: Every 14, 21, or 28 days
mOS: Median overall survival
mPFS: Median progression-free survival
HR: Hazard ratio
CI: Confidence interval
ORR: Objective response rate
DCR: Disease control rate
mDoR: Median duration of response
RECIST: Response Evaluation Criteria in Solid Tumors
CR: Complete response
PR: Partial response
SD: Stable disease
PD: Progressive disease
mo: Months
NR: Not reached/Not reported
NA/N/A: Not available/Not applicable
TEAEs: Treatment-emergent adverse events
irAEs: Immune-related adverse events
G3+: Grade 3 or higher
Neutropenia: Abnormally low count of neutrophils
Anemia: Low hemoglobin level
Thrombocytopenia: Low blood platelet count
Pneumonitis: Inflammation of lung tissue
Hepatitis: Inflammation of the liver
Neuropathy: Nerve damage or dysfunction
PRISMA: Preferred Reporting Items for Systematic Reviews and Meta-Analyses
CNKI: China National Knowledge Infrastructure
JADAD Scale: Quality scale evaluating randomization, concealment, blinding, and attrition
MINORS Scale: Methodological Index for Non-Randomized Studies
Unbiased: Objectivity of endpoint evaluation or blinded assessment.
