# Supplementary figures and images for: Advancing precision immunotherapy in advanced pancreatic cancer: a systematic review and meta-analysis of first-line ICI-based combinations
Source: Front Immunol. 2026 Jul 10;17:1855859. doi: 10.3389/fimmu.2026.1855859 (PMC13396027; doi:10.3389/fimmu.2026.1855859)

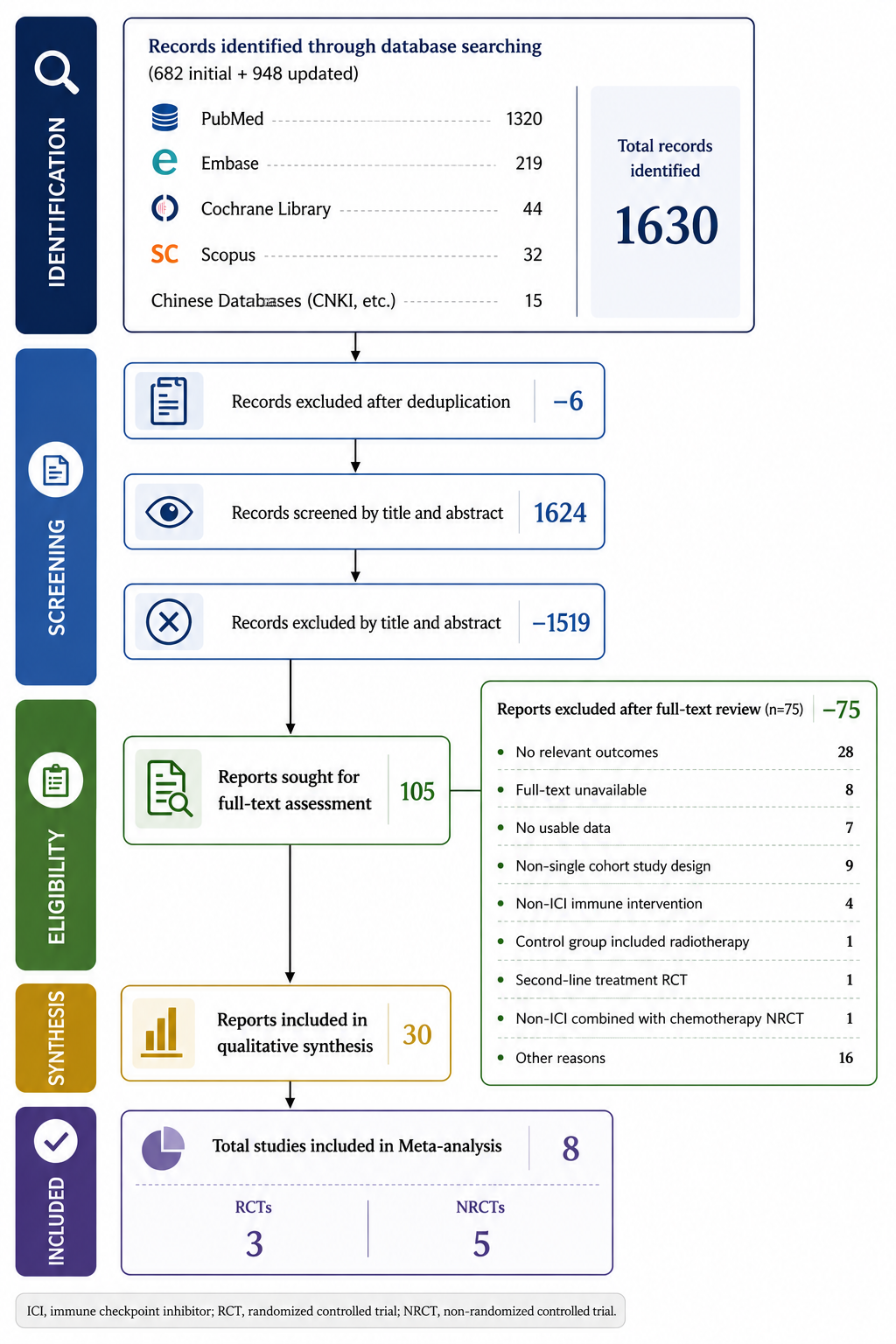

Supplement: Supplementary Figure 1 — PRISMA flow diagram illustrating the systematic identification, screening, eligibility, and inclusion process of randomized controlled trials for the meta-analysis. [file Image1.png]

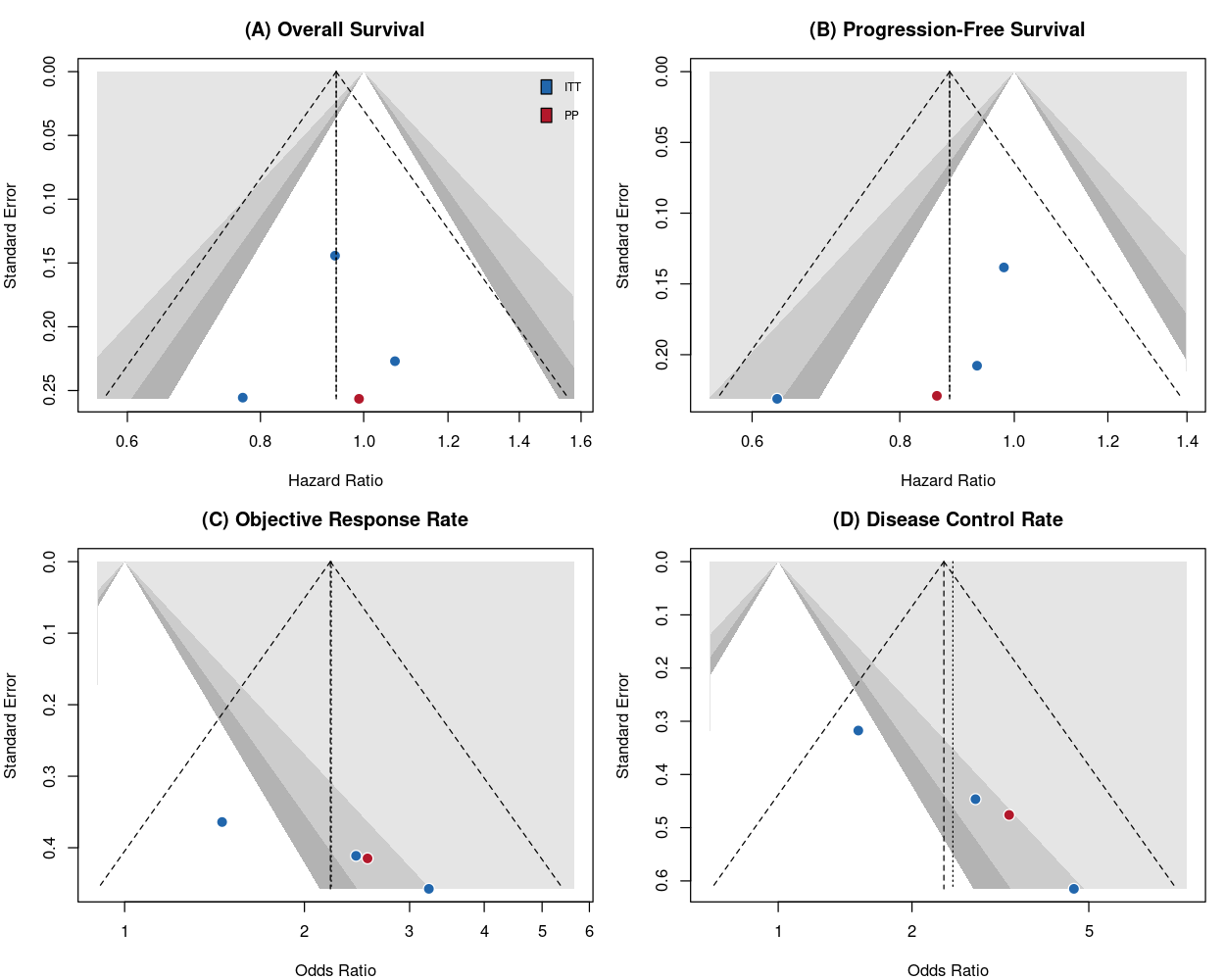

Supplement: Supplementary Figure 2 — Publication bias and small-study effect analysis of clinical outcomes, including contour-enhanced funnel plots for assessment of publication bias (Panels A–D) in RCTs of first-line ICI-based combination therapies. [file Image2.tiff]

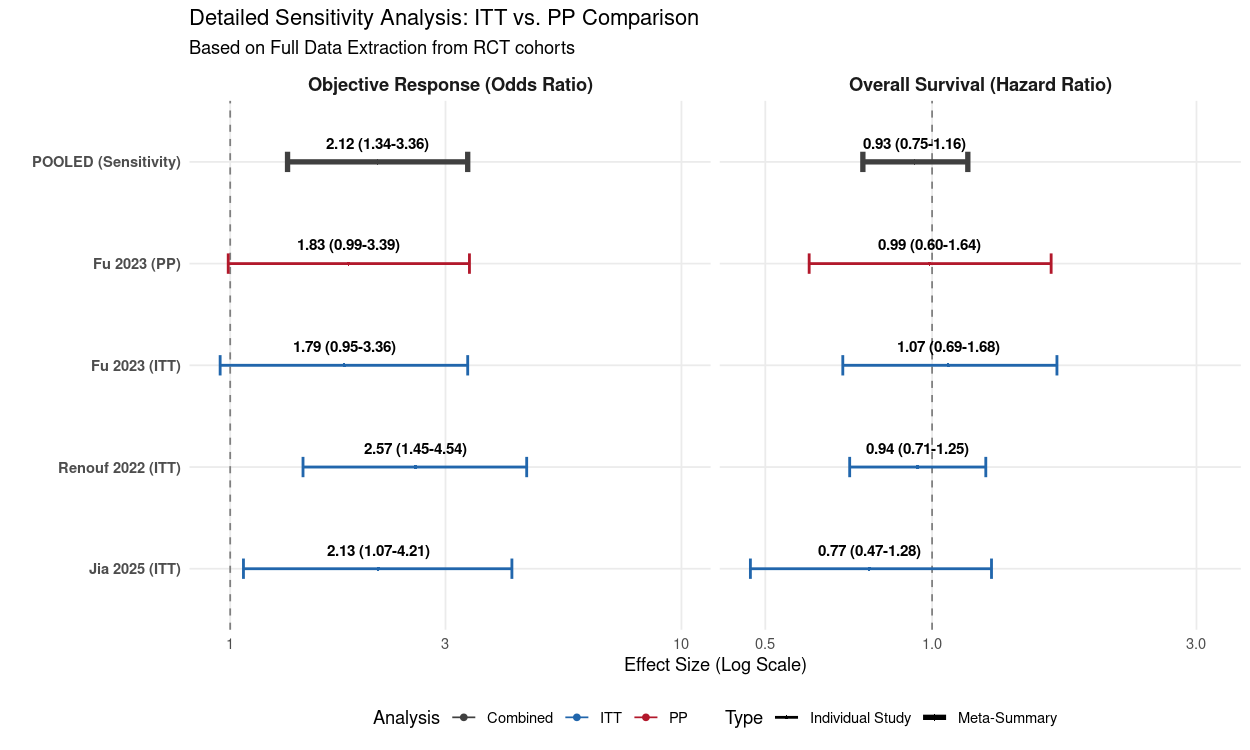

Supplement: Supplementary Figure 3 — Leave-one-out sensitivity analysis for the primary outcomes (ORR and OS) in RCTs of first-line ICI-based combination therapies. [file Image3.tiff]

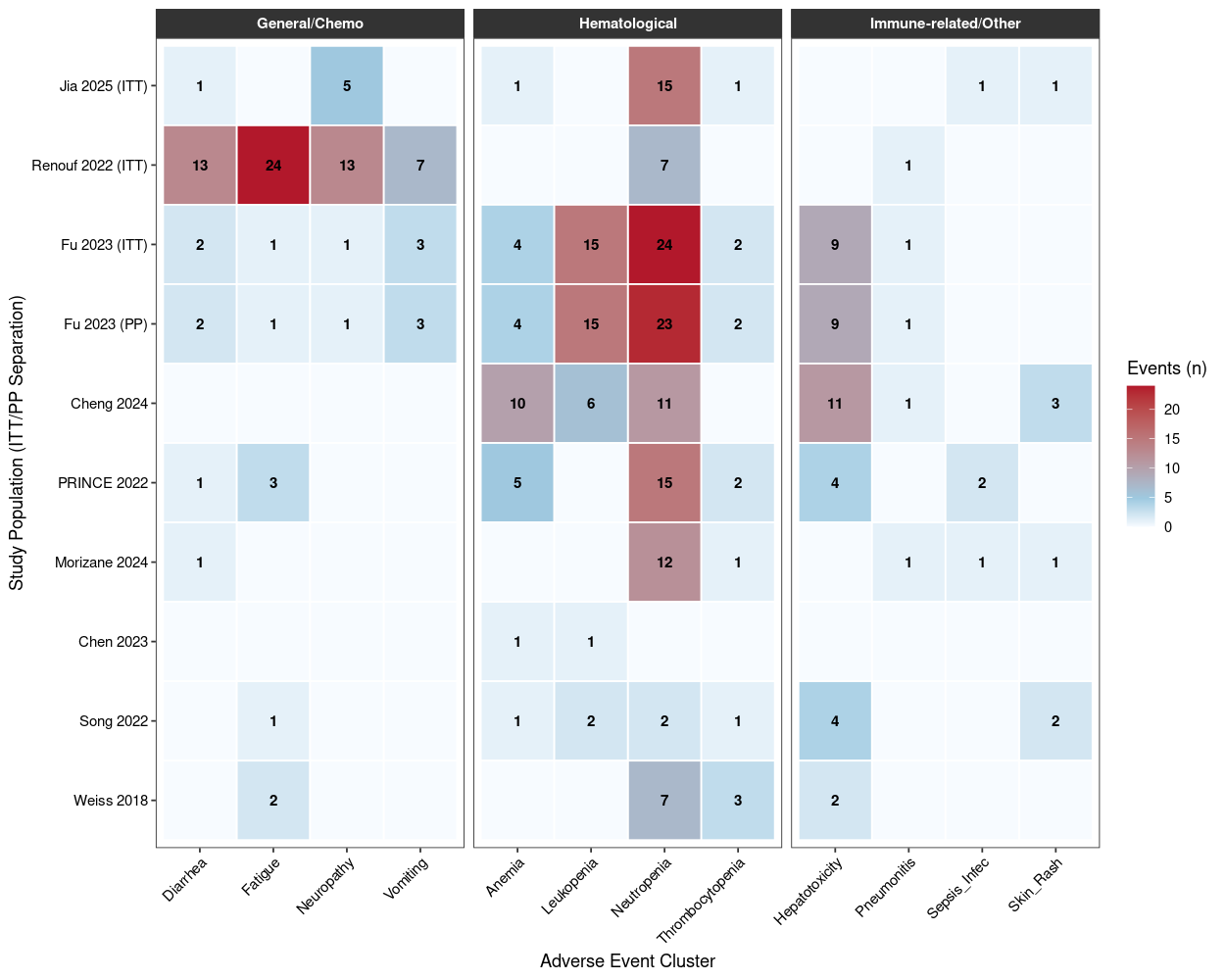

Supplement: Supplementary Figure 4 — Heatmap of Grade 3 or higher adverse events (General/Chemo, Hematological, and Immune-related) across included RCTs of first-line ICI-based combination therapies. [file Image4.tiff]

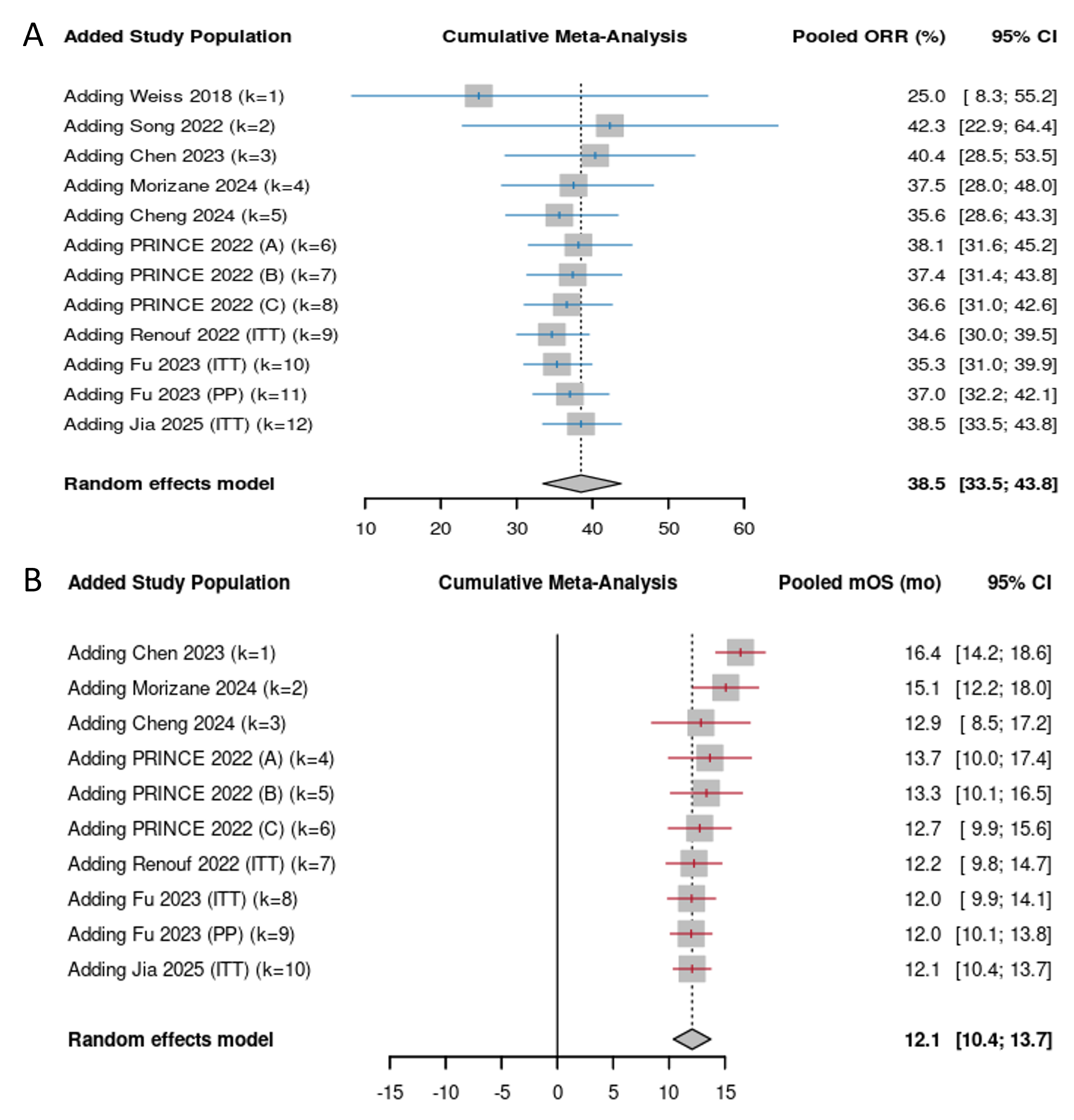

Supplement: Supplementary Figure 5 — Cumulative meta-analysis of clinical outcomes over publication years in RCTs of first-line ICI-based combination therapies. [file Image5.tif]
