# Supplementary material for: Advancing precision immunotherapy in advanced pancreatic cancer: a systematic review and meta-analysis of first-line ICI-based combinations
Source: Front Immunol. 2026 Jul 10;17:1855859. doi: 10.3389/fimmu.2026.1855859 (PMC13396027; doi:10.3389/fimmu.2026.1855859)
Supplement: Supplementary file 6 [file Table1.docx]

Supplementary Material

1. Appendix Table 1. Methodological quality assessment using JADAD and MINORS scales.
2. JADAD Scale for Randomized Controlled Trials (RCTs)

| **Included Study** | **Randomization** | **Allocation Concealment** | **Blinding** | **Withdrawals and Dropouts** | **Total Score** | **Quality Level** |
| --- | --- | --- | --- | --- | --- | --- |
| **Renouf 2022** | 1 | 2 | 0 | 1 | **4** | High Quality |
| **Fu 2023** | 2 | 2 | 0 | 1 | **5** | High Quality |
| **Jia 2025** | 2 | 1 | 0 | 1 | **4** | High Quality |

1. MINORS Scale for Non-Randomized and Single-arm Studies

| **Included Study** | **Aim** | **Patients** | **Prospective** | **Endpoints** | **Unbiased** | **Follow-up** | **Loss <5%** | **Size** | **Total Score** |
| --- | --- | --- | --- | --- | --- | --- | --- | --- | --- |
| **Weiss 2018** | 2 | 1 | 2 | 2 | 1 | 2 | 2 | 1 | 13 |
| **Song 2022** | 2 | 1 | 0 | 2 | 2 | 2 | 2 | 0 | 11 |
| **Chen 2023** | 2 | 1 | 0 | 2 | 2 | 2 | 2 | 0 | 11 |
| **Morizane 2024** | 2 | 2 | 2 | 2 | 2 | 2 | 2 | 2 | 16 |
| **Cheng 2024** | 2 | 2 | 2 | 2 | 2 | 2 | 2 | 2 | 16 |

Note: ADAD Scale: Evaluation dimensions include randomization (0-2 points), concealed assignment (0-2 points), blinding (0-2 points; this item is scored as 0 points since most studies in this group were open-label), and withdrawal or loss to follow-up (0-1 points). A total score ≥3 points is considered high-quality.

MINORS Scale: The revised non-randomized study methodological evaluation index. It evaluates single-arm studies using 8 indicators, with each item scored 0-2 points (0: not reported; 1: reported but with insufficient information; 2: reported in detail and meets requirements), totaling 16 points.

High Quality: Refers to methodologically rigorous high-quality literature.

Unbiased: Refers to the objectivity of endpoint evaluation or blinded assessment.

Appendix Table 2. Detailed search strategy for each electronic database.

| **Phase** | **Details** | **Number**  **(n)** |
| --- | --- | --- |
| **Identification** | **Total records identified through database searching** (682 initial + 948 updated)  • PubMed: 1320  • Embase: 219  • Cochrane Library: 44  • Scopus: 32  • Chinese Databases (CNKI, etc.): 15 | **1630** |
| **Screening** | Records excluded after deduplication | -6 |
|  | **Records screened by title and abstract** | **1624** |
|  | Records excluded by title and abstract | -1519 |
| **Eligibility** | **Reports sought for full-text assessment** | **105** |
|  | **Reports excluded after full-text review** (n=75)  • No relevant outcomes: 28  • Full-text unavailable: 8  • No usable data: 7  • Non-single cohort study design: 9  • Non-ICI immune intervention: 4  • Control group included radiotherapy: 1  • **Second-line treatment RCT: 1**  • **Non-ICI combined with chemotherapy NRCT: 1**  • Other reasons: 16 | **-75** |
| **Synthesis** | Reports included in qualitative synthesis | **30** |
| **Included** | **Total studies included in Meta-analysis**  • **RCTs: 3**  • **NRCTs: 5** | **8** |

**Note:Abbreviations**: **PRISMA**, Preferred Reporting Items for Systematic Reviews and Meta-Analyses; **CNKI**, China National Knowledge Infrastructure; **ICI**, Immune Checkpoint Inhibitor; **RCT**, Randomized Controlled Trial; **NRCT**, Non-Randomized Controlled Trial.

**Search Strategy**: The literature search was conducted across multiple electronic databases including PubMed, Embase, Cochrane Library, and Scopus, supplemented by major Chinese biomedical databases.

**Exclusion Criteria**: Full-text reports were excluded if they failed to meet the predefined inclusion criteria, such as studies focusing on second-line therapy or those utilizing non-ICI based immunotherapy combinations.

Appendix Table 3. Supplementary baseline clinical characteristics of the enrolled patients.

| **Study ID** | **Group** | **DOI / PMID** | **Country** | **Median Age**  **(Range)** | **ECOG PS 0/1**  **(n)** | **Primary Site**  **(Head / Body & Tail)** |
| --- | --- | --- | --- | --- | --- | --- |
| **Renouf 2022** | TG | 10.1038/s41467-022-32591-8 | Canada | 64 (29–81) | 49 / 70 | **NR** |
|  | CG |  |  | 65 (42–84) | 24 / 34 | **NR** |
| **Fu 2023** | TG | 10.1245/s10434-023-13383-w | China | 61 (44–74) | 17 / 38 | 16 / 39 (29.1% / 70.9%) |
|  | CG |  |  | 62 (45–73) | 18 / 37 | 20 / 34 (36.3% / 61.8%) |
| **Jia 2025** | TG | 10.1038/s41392-025-02441-2 | China | 59.0 (36.0–75.0) | 10 / 35 | 18 / 27 (40.0% / 60.0%) |
|  | CG |  |  | 59.0 (39.0–74.0) | 11 / 34 | 19 / 26 (42.2% / 57.8%) |
| **Weiss 2018** | TG | 10.1007/s10637-017-0525-1 | USA | 55 (46–66) | 8 / 9 | **NR** |
| **Song 2022** | TG | 10.2217/imt-2022-0196 | China | 62 (38–80) | 6 / 12 | 7 / 11 (38.9% / 61.1%) |
| **Chen 2023** | TG | 10.2147/OTT.S427942 | China | 64 (46–77) | 8 / 19 | 6 / 21 (22.2% / 77.8%) |
| **Morizane 2024** | TG | 10.1038/s44276-023-00028-4 | Japan | 59.0 (39–75) | 16 / 15 | 9 / 22 (29.0% / 71.0%)* |
| **Cheng 2024** | TG | 10.1038/s41392-024-02031-8 | China | 57 (43–71) | 20 / 52 | 36 / 36 (50.0% / 50.0%) |

**Note:ECOG PS**: Eastern Cooperative Oncology Group Performance Status；**DOI**: Digital Object Identifier（；**B&T**: Body and Tail；**TG/CG**: Treatment Group / Control Group。

Appendix Table 4. Detailed treatment protocols and dose modifications.

| **Study ID** | **Treatment Regimen**  **(Drugs)** | **Specific Dosage and Route of Administration** | **Cycle Duration** | **Dose Modification / Management** |
| --- | --- | --- | --- | --- |
| **Renouf 2022** | Durvalumab + Tremelimumab + Gem + nab-P | Dur: 1500mg (D1); Tre: 75mg (D1); Gem: 1000mg/m² (D1, 8, 15); nab-P: 125mg/m² (D1, 8, 15) [IV] | 28 days | Discontinuation for Grade 3+ irAEs; dose reduction allowed for hematologic toxicity. |
| **Fu 2023** | Sintilimab + mFOLFIRINOX | Sin: 200mg (q3w); mFFX: Oxaliplatin 68mg/m², Irinotecan 135mg/m², Leucovorin 400mg/m², 5-FU 2400mg/m² (46h) [IV] | 14 days | Dose reduction allowed for Grade 3/4 neutropenia or thrombocytopenia. |
| **Jia 2025** | NASCA (Fruquintinib + Camrelizumab + nab-P + S-1) | Fru: 200mg (PO, qd); Cam: 200mg (IV, D1); nab-P: 125mg/m² (IV, D1, 8); S-1: 40mg (PO, bid, D1-14) | 21 days | Complex adjustments for non-hematologic toxicities (G3+); 33.3% required discontinuation. |
| **Weiss 2018** | Pembrolizumab + Gem + nab-P | Pem: 2mg/kg (q3w); Gem: 1000mg/m²; nab-P: 125mg/m² (D1, 8) [IV] | 21 days | No dose reduction for Pembrolizumab; reduction allowed for chemotherapy components. |
| **Song 2022** | PD-1 Inhibitor + nab-P + Gem | PD-1: 200mg (q3w); nab-P: 135mg/m²; Gem: 1000mg/m² (D1, 8) [IV] | 21 days | Modified based on clinical tolerability; 27.8% required treatment discontinuation. |
| **Chen 2023** | PD-1 Inhibitor + Gem + nab-P | PD-1: Standard dose (q3w); Gem: 1000mg/m²; nab-P: 125mg/m² (D1, 8) [IV] | 21 days | Dose delay permitted for Grade 3 hematologic events; no treatment-related deaths reported. |
| **Morizane 2024** | Nivolumab + mFOLFIRINOX | Nivo: 480mg (q4w); mFFX: Oxaliplatin 85mg/m², Irinotecan 150mg/m², Leucovorin 200mg/m², 5-FU 2400mg/m² [IV] | 14 days | Stringent criteria for dose delays due to irAEs (48.4% any grade irAEs). |
| **Cheng 2024** | Toripalimab + nab-P + Gem | Tori: 240mg (D1); nab-P: 125mg/m² (D1, 8); Gem: 1000mg/m² (D1, 8) [IV] | 21 days | Protocol-defined reduction for neurotoxicity and G3+ AEs; maintenance with Tori allowed after 6 cycles. |

**Note:Dur**: Durvalumab; **Tre**: Tremelimumab; **Sin**: Sintilimab; **Cam**: Camrelizumab; **Pem**: Pembrolizumab; **Nivo**: Nivolumab; **Tori**: Toripalimab; **Fru**: Fruquintinib.

**Gem**: Gemcitabine; **nab-P**: nab-paclitaxel; **mFOLFIRINOX/mFFX**: modified Fluorouracil, Leucovorin, Oxaliplatin, and Irinotecan.

**IV**: Intravenous; **PO**: Per os (Oral).

**q28d/q21d/q14d**: Every 28, 21, or 14 days.

Appendix Table 5. Table. Grade ≥ 3 Adverse Events and Safety Outcomes

| **Adverse Events**  **(Grade ≥ 3)** | **Renouf 2022**  **(TG/CG)** | **Fu 2023**  **(TG/CG)** | **Jia 2025**  **(TG/CG)** | **Weiss 2018**  **(TG)** | **Song 2022**  **(TG)** | **Chen 2023**  **(TG)** | **Morizane 2024**  **(TG)** | **Cheng 2024**  **(TG)** |
| --- | --- | --- | --- | --- | --- | --- | --- | --- |
| **N_Safety** | **119 / 58** | **55 / 55** | **45 / 45** | **17** | **18** | **27** | **31** | **72** |
| **Hematologic, n (%)** |  |  |  |  |  |  |  |  |
| G3+ Neutropenia | 7 (5.9) / 4 (6.9) | 24 (43.6) / 22 (40.0) | 15 (33.3) / 16 (35.6) | 7 (41.2) | 2 (11.1) | 0 (0) | 12 (38.7) | 11 (15.3) |
| G3+ Anemia | NR / NR | 4 (7.3) / 3 (5.5) | 1 (2.2) / 1 (2.2) | 3 (17.6) | 1 (5.6) | 1 (3.7) | 0 (0) | 10 (13.9) |
| G3+ Thrombocytopenia | NR / NR | 2 (3.6) / 2 (3.6) | 1 (2.2) / 17 (37.8) | 1 (5.9) | 1 (5.6) | 0 (0) | 0 (0) | NA |
| **Non-hematologic, n (%)** |  |  |  |  |  |  |  |  |
| G3+ Fatigue | 24 (20.2) / 12 (20.7) | 1 (1.8) / 2 (3.6) | 0 (0) / 2 (4.4) | 2 (11.8) | 1 (5.6) | 0 (0) | 0 (0) | NA |
| G3+ Vomiting | 7 (5.9) / 2 (3.4) | 3 (5.5) / 3 (5.5) | 0 (0) / 0 (0) | 0 (0) | 0 (0) | 0 (0) | 0 (0) | NA |
| G3+ Neuropathy | 13 (10.9) / 7 (12.1) | 1 (1.8) / 0 (0) | 5 (11.1) / 0 (0) | 0 (0) | 0 (0) | 0 (0) | 0 (0) | NA |
| **Specific irAEs (G3+), n (%)** |  |  |  |  |  |  |  |  |
| G3+ Pneumonitis | 1 (0.8) / 0 (0) | 1 (1.8) / 0 (0) | 0 (0) / 0 (0) | 0 (0) | 0 (0) | 0 (0) | 1 (3.2) | 1 (1.4) |
| G3+ Hepatitis | NR / NR | 1 (1.8) / 0 (0) | 0 (0) / 0 (0) | 1 (5.9) | 0 (0) | 0 (0) | 0 (0) | 1 (1.4) |
| G3+ Colitis | 1 (0.8) / 0 (0) | 0 (0) / 0 (0) | 0 (0) / 0 (0) | 0 (0) | 0 (0) | 0 (0) | 0 (0) | NA |
| G3+ Pancreatitis | NR / NR | 1 (1.8) / 0 (0) | 0 (0) / 0 (0) | 0 (0) | 0 (0) | 0 (0) | 0 (0) | NA |
| **Overall Outcomes, n (%)** |  |  |  |  |  |  |  |  |
| Discontinuation (AEs) | NR / NR | 3 (5.5) / 0 (0) | 15 (33.3) / 10 (22.2) | 0 (0) | 5 (27.8) | 0 (0) | 1 (3.2) | 5 (6.9) |
| AE-related Death | 2 (1.7) / 2 (3.4) | 0 (0) / 0 (0) | 0 (0) / 0 (0) | 0 (0) | 0 (0) | 0 (0) | 0 (0) | 0 (0) |

**Note:G3+**: Grade 3 or higher；**irAEs**: Immune-related Adverse Events；**TG/CG**: Treatment Group / Control Group；**NA**: Not Available；**Neuropathy**:

**Appendix Table 6. Study classification and analysis decision.**

| **Order** | **Study** | **Design** | **Phase** | **Randomized** | **Country** | **RCT HR pooling** | **Single-arm use** | **Caution** |
| --- | --- | --- | --- | --- | --- | --- | --- | --- |
| 1 | Weiss 2018 | NRCT/single-arm | Phase Ib/II | No | USA | No | Yes | Efficacy first-line subset N=12 treated / 11 response-evaluable; safety not cleanly limited to first-line subset. |
| 2 | Song 2022 | NRCT/retrospective case series | Case series/retrospective | No | China | No | Yes | Exact LAPC vs mPDAC split not extractable; no reported aggregate mOS/mPFS. |
| 3 | Chen 2023 | NRCT/retrospective | Real-world single-center | No | China | No | Yes | Contains 16 locally advanced and 11 liver-metastatic patients; stage mix must be flagged. |
| 4 | Morizane 2024 | NRCT/prospective single-arm | Phase 2 | No | Japan | No | Yes | Full analysis set and safety set both N=31; metastatic-only. |
| 5 | Cheng 2024 | NRCT/prospective single-arm | Phase Ib/II | No | China | No | Yes | Contains LAPC N=6 and mPDAC N=66; ITT/safety N=72. |
| 6 | PRINCE 2022 | RCT/multi-arm randomized phase 2 | Phase 1b/2 randomized | Yes | USA/multicenter | No for chemo-controlled HR analysis | Yes for ICI-containing arms only | No chemotherapy-alone control; primary endpoint used historical 1-year OS. Sotigalimab-only arm is CD40 agonist + che... |
| 7 | Renouf 2022 PA.7 | RCT | Phase II | Yes | Canada | Yes | Yes, if arm-level response/survival landscape needed | Control safety population N=58 while randomized/ITT control N=61; HR CIs are 90% CIs. |
| 8 | Fu 2023 CISPD3 | RCT | Phase II | Yes | China | Yes | Yes, if arm-level response/survival landscape needed | ITT N=55/55, PP N=45/46, response-evaluable N=44/46, safety N=53/54; OS/PFS HRs corrected from uploaded paper. |
| 9 | Jia 2025 NASCA | RCT | Phase Ib/II randomized | Yes | China | Yes | Yes, if arm-level response/survival landscape needed | Contains LAPC N=8/9 and mPDAC N=37/36; both arms ITT/safety N=45. |

**Appendix Table 7. Arm-level regimen metadata and denominators.**

| **Study** | **Arm** | **Role** | **Regimen** | **ICI class** | **Chemo backbone** | **Anti-angio** | **N rand** | **N treated** | **N efficacy** | **N safety** |
| --- | --- | --- | --- | --- | --- | --- | --- | --- | --- | --- |
| Weiss 2018 | Weiss_TG | single-arm | Pembrolizumab + gemcitabine + nab-paclitaxel | PD-1 | Gem+nab-P | No | NR/NA | 17 | 12 | 17 |
| Song 2022 | Song_TG | single-arm | PD-1 inhibitor + gemcitabine + albumin-bound paclitaxel | PD-1 mixed | Gem+nab-P/AG | No | NR/NA | 18 | 18 | 18 |
| Chen 2023 | Chen_TG | single-arm | PD-1 inhibitor + gemcitabine + nab-paclitaxel | PD-1 mixed | Gem+nab-P | No | NR/NA | 27 | 27 | 27 |
| Morizane 2024 | Morizane_TG | single-arm | Nivolumab + modified FOLFIRINOX | PD-1 | mFOLFIRINOX | No | NR/NA | 31 | 31 | 31 |
| Cheng 2024 | Cheng_TG | single-arm | Toripalimab + gemcitabine + nab-paclitaxel | PD-1 | Gem+nab-P | No | NR/NA | 72 | 72 | 72 |
| PRINCE 2022 | PRINCE_Nivo_Chemo | randomized arm | Nivolumab + gemcitabine + nab-paclitaxel | PD-1 | Gem+nab-P | No | 37 | 34 | 34 | 36 |
| PRINCE 2022 | PRINCE_Sotiga_Chemo | randomized arm / non-ICI | Sotigalimab + gemcitabine + nab-paclitaxel | Non-ICI immunomodulator | Gem+nab-P | No | 31 | 30 | 36 | 37 |
| PRINCE 2022 | PRINCE_Sotiga_Nivo_Chemo | randomized arm | Sotigalimab + nivolumab + gemcitabine + nab-paclitaxel | PD-1 + CD40 agonist | Gem+nab-P | No | 31 | 27 | 35 | 35 |
| Renouf 2022 PA.7 | Renouf_TG | experimental | Durvalumab + tremelimumab + gemcitabine + nab-paclitaxel | PD-L1 + CTLA-4 | Gem+nab-P | No | 119 | 119 | 119 | 119 |
| Renouf 2022 PA.7 | Renouf_CG | control | Gemcitabine + nab-paclitaxel | None | Gem+nab-P | No | 61 | 58 | 61 | 58 |
| Fu 2023 CISPD3 | Fu_TG | experimental | Sintilimab + modified FOLFIRINOX | PD-1 | mFOLFIRINOX | No | 55 | 53 | 44 | 53 |
| Fu 2023 CISPD3 | Fu_CG | control | Modified FOLFIRINOX | None | mFOLFIRINOX | No | 55 | 54 | 46 | 54 |
| Jia 2025 NASCA | Jia_TG | experimental | Surufatinib + camrelizumab + nab-paclitaxel + S-1 | PD-1 | nab-P + S-1 | Surufatinib | 45 | 45 | 45 | 45 |
| Jia 2025 NASCA | Jia_CG | control | Nab-paclitaxel + gemcitabine | None | Gem+nab-P | No | 45 | 45 | 45 | 45 |

**Appendix Table 8. LAPC/mPDAC stage extraction and liver metastasis counts.**

| **Study** | **Arm** | **LAPC n** | **mPDAC n** | **Recurrent n** | **Unknown stage n** | **Liver met n** | **Note** |
| --- | --- | --- | --- | --- | --- | --- | --- |
| Weiss 2018 | Weiss_TG | 0 | 17 | NR/NA | NR/NA | NR/NA | All histologically confirmed metastatic PDAC; first-line chemo-naive subset N=12 |
| Song 2022 | Song_TG | NR/NA | NR/NA | NR/NA | 18 | 10 | Advanced stage III-IV; exact LAPC/mPDAC not extractable |
| Chen 2023 | Chen_TG | 16 | 11 | NR/NA | NR/NA | 11 | Text states 16 locally advanced and 11 had liver metastasis; table lists no metastasis=3, lymph node=23. |
| Morizane 2024 | Morizane_TG | 0 | 31 | NR/NA | NR/NA | 20 | Primary site body and tail reported separately; do not sum with head as mutually exclusive without checking. |
| Cheng 2024 | Cheng_TG | 6 | 66 | NR/NA | NR/NA | 51 | ITT efficacy and safety among all 72 eligible patients who received at least one cycle. |
| PRINCE 2022 | PRINCE_Nivo_Chemo | 0 | 34 | NR/NA | NR/NA | NR/NA | Efficacy N includes randomized/dosed phase 2; safety N=36. De novo stage IV:27/34. |
| PRINCE 2022 | PRINCE_Sotiga_Chemo | 0 | 36 | NR/NA | NR/NA | NR/NA | Non-ICI CD40 agonist arm; de novo stage IV:27/36. |
| PRINCE 2022 | PRINCE_Sotiga_Nivo_Chemo | 0 | 35 | NR/NA | NR/NA | NR/NA | ICI+CD40 agonist; de novo stage IV:26/35. |
| Renouf 2022 PA.7 | Renouf_TG | 0 | 119 | NR/NA | NR/NA | NR/NA | mPDAC only; liver metastasis not reported in Table 1. |
| Renouf 2022 PA.7 | Renouf_CG | 0 | 61 | NR/NA | NR/NA | NR/NA | mPDAC only; safety denominator N=58 because 3 control patients not treated. |
| Fu 2023 CISPD3 | Fu_TG | 0 | 52 | 3 | NR/NA | 44 | Metastatic/recurrent; line of chemotherapy first-line 52, second-line 3. Response-evaluable N=44; safety N=53. |
| Fu 2023 CISPD3 | Fu_CG | 0 | 50 | 5 | NR/NA | 39 | Metastatic/recurrent; line of chemotherapy first-line 50, second-line 5. Response-evaluable N=46; safety N=54. |
| Jia 2025 NASCA | Jia_TG | 8 | 37 | NR/NA | NR/NA | NR/NA | Liver metastasis count not extracted from uploaded PDF text; stage split from Table 1. |
| Jia 2025 NASCA | Jia_CG | 9 | 36 | NR/NA | NR/NA | NR/NA | Liver metastasis count not extracted from uploaded PDF text; stage split from Table 1. |

**Appendix Table 9. R-ready comparative HR dataset.**

| **Study** | **Endpoint** | **Set** | **TG** | **CG** | **HR** | **CI low** | **CI high** | **CI level** | **P** | **TG median** | **CG median** | **Note** |
| --- | --- | --- | --- | --- | --- | --- | --- | --- | --- | --- | --- | --- |
| Renouf 2022 PA.7 | OS | ITT | Renouf_TG | Renouf_CG | 0.94 | 0.71 | 1.25 | 90 | 0.72 | 9.80 | 8.80 | stratified Cox/log-rank; HR CI is 90% |
| Renouf 2022 PA.7 | PFS | ITT | Renouf_TG | Renouf_CG | 0.98 | 0.75 | 1.29 | 90 | 0.91 | 5.50 | 5.40 | stratified Cox/log-rank; HR CI is 90% |
| Fu 2023 CISPD3 | OS | ITT | Fu_TG | Fu_CG | 1.07 | 0.69 | 1.68 | 95 | >0.05 | 10.90 | 10.80 | CORRECTED from uploaded paper; no OS benefit |
| Fu 2023 CISPD3 | PFS | ITT | Fu_TG | Fu_CG | 0.93 | 0.62 | 1.40 | 95 | >0.05 | 5.90 | 5.70 | CORRECTED from uploaded paper; figure OCR may look like 1.93 but abstract/text show 0.93 with CI 0.62-1.40 |
| Jia 2025 NASCA | OS | ITT | Jia_TG | Jia_CG | 0.77 | 0.47 | 1.28 | 95 | 0.32 | 13 | 11 | Cox/log-rank |
| Jia 2025 NASCA | PFS | ITT | Jia_TG | Jia_CG | 0.63 | 0.40 | 0.99 | 95 | 0.04 | 7.90 | 5.30 | Cox/log-rank |

**Appendix Table 10. R-ready response-rate dataset with explicit denominators and CIs.**

| **Study** | **Arm** | **Set** | **N** | **CR** | **PR** | **SD** | **PD** | **NE** | **ORR n** | **ORR %** | **ORR CI low** | **ORR CI high** | **DCR n** | **DCR %** | **DCR CI low** | **DCR CI high** | **CI method** |
| --- | --- | --- | --- | --- | --- | --- | --- | --- | --- | --- | --- | --- | --- | --- | --- | --- | --- |
| Weiss 2018 | Weiss_TG | chemo-naive treated/conservative | 12 | 0 | 3 | 8 | 0 | 1 | 3 | 25 | 8.89 | 53.23 | 11 | 91.67 | 64.61 | 98.51 | Wilson 95% CI estimated for missing rate CIs |
| Song 2022 | Song_TG | all treated | 18 | 1 | 9 | 5 | 3 | 0 | 10 | 55.56 | 33.72 | 75.44 | 15 | 83.33 | 60.78 | 94.16 | Wilson 95% CI estimated for missing rate CIs |
| Chen 2023 | Chen_TG | all treated | 27 | 0 | 10 | 10 | 7 | 0 | 10 | 37.04 | 21.53 | 55.77 | 20 | 74.07 | 55.32 | 86.83 | Wilson 95% CI estimated for missing rate CIs |
| Morizane 2024 | Morizane_TG | full analysis set | 31 | 0 | 10 | 12 | 7 | 2 | 10 | 32.30 | 18.70 | 48.50 | 22 | 71 | 53.41 | 83.90 | reported Clopper-Pearson 90% CI for ORR; DCR CI estimated separately if needed |
| Cheng 2024 | Cheng_TG | ITT | 72 | 1 | 23 | 41 | 7 | 0 | 24 | 33.30 | 23.53 | 44.82 | 65 | 90.30 | 81.26 | 95.21 | Wilson 95% CI estimated for missing rate CIs |
| PRINCE 2022 | PRINCE_Nivo_Chemo | efficacy population | 34 | NR/NA | NR/NA | 8 | NR/NA | NR/NA | 17 | 50 | 32 | 68 | 25 | 74 | 56 | 87 | reported 95% CI for rates; response component counts partly reconstructed from ORR/DCR |
| PRINCE 2022 | PRINCE_Sotiga_Nivo_Chemo | efficacy population | 35 | NR/NA | NR/NA | 13 | NR/NA | NR/NA | 11 | 31 | 17 | 49 | 24 | 69 | 51 | 83 | reported 95% CI for rates; response component counts partly reconstructed from ORR/DCR |
| Renouf 2022 PA.7 | Renouf_TG | ITT | 119 | 0 | 36 | NR/NA | NR/NA | NR/NA | 36 | 30.30 | 22.72 | 39.02 | 84 | 70.60 | 61.86 | 78.03 | Wilson 95% CI estimated for missing rate CIs |
| Renouf 2022 PA.7 | Renouf_CG | ITT | 61 | 0 | 14 | NR/NA | NR/NA | NR/NA | 14 | 23 | 14.19 | 34.91 | 35 | 57.40 | 44.90 | 68.98 | Wilson 95% CI estimated for missing rate CIs |
| Fu 2023 CISPD3 | Fu_TG | response-evaluable | 44 | 1 | 21 | 15 | 7 | 0 | 22 | 50 | 34.60 | 65.40 | 37 | 84.10 | 72.80 | 95.30 | reported 95% CI in response-evaluable population |
| Fu 2023 CISPD3 | Fu_CG | response-evaluable | 46 | 0 | 11 | 22 | 13 | 0 | 11 | 23.90 | 11.10 | 36.70 | 33 | 71.70 | 58.20 | 85.30 | reported 95% CI in response-evaluable population |
| Jia 2025 NASCA | Jia_TG | ITT | 45 | 0 | 23 | 18 | 4 | 0 | 23 | 51.10 | 35.80 | 66.30 | 41 | 91.10 | 78.80 | 97.50 | reported Clopper-Pearson exact 95% CI |
| Jia 2025 NASCA | Jia_CG | ITT | 45 | 0 | 11 | 29 | 5 | 0 | 11 | 24.40 | 12.90 | 39.50 | 40 | 88.90 | 75.90 | 96.30 | reported Clopper-Pearson exact 95% CI |

For rate meta-analysis, use event counts and denominators instead of rounded percentages. Estimated CIs are for transparent reporting only.

**Appendix Table 11. R-ready Grade 3 or higher safety dataset.**

| **Study** | **Arm** | **Safety N** | **Denominator** | **Adverse event** | **Event n** | **Event %** | **Event status** |
| --- | --- | --- | --- | --- | --- | --- | --- |
| Weiss 2018 | Weiss_TG | 17 | safety population/treated | Treatment-related death | 0 | 0 | reported_zero |
| Song 2022 | Song_TG | 18 | safety population/treated | Thrombocytopenia | 2 | 11.11 | reported |
| Chen 2023 | Chen_TG | 27 | safety population/treated | Neutropenia | 0 | 0 | reported_zero |
| Chen 2023 | Chen_TG | 27 | safety population/treated | Thrombocytopenia | 0 | 0 | reported_zero |
| Chen 2023 | Chen_TG | 27 | safety population/treated | Anemia | 1 | 3.70 | reported |
| Morizane 2024 | Morizane_TG | 31 | safety population/treated | Peripheral sensory neuropathy | 0 | 0 | reported_zero |
| Morizane 2024 | Morizane_TG | 31 | safety population/treated | Interstitial lung disease/pneumonitis | 1 | 3.23 | reported |
| Morizane 2024 | Morizane_TG | 31 | safety population/treated | Drug-related death within 30-day safety window | 0 | 0 | reported_zero |
| Cheng 2024 | Cheng_TG | 72 | safety population/treated | Neutropenia | 11 | 15.28 | reported |
| Cheng 2024 | Cheng_TG | 72 | safety population/treated | Anemia | 10 | 13.89 | reported |
| Cheng 2024 | Cheng_TG | 72 | safety population/treated | Interstitial pneumonia/pneumonitis | 1 | 1.39 | reported |
| Cheng 2024 | Cheng_TG | 72 | safety population/treated | Treatment-related death | 0 | 0 | reported_zero |
| PRINCE 2022 | PRINCE_Nivo_Chemo | 36 | safety population/treated | AE leading to discontinuation of all study drugs | 6 | 16.67 | reported |
| PRINCE 2022 | PRINCE_Nivo_Chemo | 36 | safety population/treated | Thrombocytopenia | 18 | 50 | reported |
| PRINCE 2022 | PRINCE_Nivo_Chemo | 36 | safety population/treated | Thrombocytopenia | 5 | 13.89 | reported |
| PRINCE 2022 | PRINCE_Sotiga_Chemo | 37 | safety population/treated | AE leading to discontinuation of all study drugs | 1 | 2.70 | reported |
| PRINCE 2022 | PRINCE_Sotiga_Chemo | 37 | safety population/treated | Thrombocytopenia | 21 | 56.76 | reported |
| PRINCE 2022 | PRINCE_Sotiga_Chemo | 37 | safety population/treated | Thrombocytopenia | 6 | 16.22 | reported |
| PRINCE 2022 | PRINCE_Sotiga_Nivo_Chemo | 35 | safety population/treated | AE leading to discontinuation of all study drugs | 1 | 2.86 | reported |
| PRINCE 2022 | PRINCE_Sotiga_Nivo_Chemo | 35 | safety population/treated | Thrombocytopenia | 22 | 62.86 | reported |
| PRINCE 2022 | PRINCE_Sotiga_Nivo_Chemo | 35 | safety population/treated | Thrombocytopenia | 7 | 20 | reported |
| Renouf 2022 PA.7 | Renouf_TG | 119 | safety population/treated | Peripheral sensory neuropathy | 13 | 10.92 | reported |
| Renouf 2022 PA.7 | Renouf_TG | 119 | safety population/treated | Grade >=3 irAE | 3 | 2.52 | reported |
| Renouf 2022 PA.7 | Renouf_TG | 119 | safety population/treated | Pneumonitis | 1 | 0.84 | reported |
| Renouf 2022 PA.7 | Renouf_TG | 119 | safety population/treated | Treatment-related death | 2 | 1.68 | reported |
| Renouf 2022 PA.7 | Renouf_CG | 58 | safety population/treated | Peripheral sensory neuropathy | 7 | 12.07 | reported |
| Renouf 2022 PA.7 | Renouf_CG | 58 | safety population/treated | Grade >=3 irAE | 0 | 0 | reported_zero |
| Renouf 2022 PA.7 | Renouf_CG | 58 | safety population/treated | Pneumonitis | 0 | 0 | reported_zero |
| Renouf 2022 PA.7 | Renouf_CG | 58 | safety population/treated | Treatment-related death | 2 | 3.45 | reported |
| Fu 2023 CISPD3 | Fu_TG | 53 | safety population/treated | Neutropenia | 31 | 58.49 | reported |
| Fu 2023 CISPD3 | Fu_TG | 53 | safety population/treated | Thrombocytopenia | 9 | 16.98 | reported |
| Fu 2023 CISPD3 | Fu_TG | 53 | safety population/treated | Anemia | 8 | 15.09 | reported |
| Fu 2023 CISPD3 | Fu_TG | 53 | safety population/treated | Grade >=3 irAE | 3 | 5.66 | reported |
| Fu 2023 CISPD3 | Fu_TG | 53 | safety population/treated | TEAE leading to death | 1 | 1.89 | reported |
| Fu 2023 CISPD3 | Fu_TG | 53 | safety population/treated | TEAE leading to discontinuation of chemotherapy | 1 | 1.89 | reported |
| Fu 2023 CISPD3 | Fu_CG | 54 | safety population/treated | Neutropenia | 24 | 44.44 | reported |
| Fu 2023 CISPD3 | Fu_CG | 54 | safety population/treated | Thrombocytopenia | 6 | 11.11 | reported |
| Fu 2023 CISPD3 | Fu_CG | 54 | safety population/treated | Anemia | 7 | 12.96 | reported |
| Fu 2023 CISPD3 | Fu_CG | 54 | safety population/treated | Grade >=3 irAE | 0 | 0 | reported_zero |
| Fu 2023 CISPD3 | Fu_CG | 54 | safety population/treated | TEAE leading to death | 0 | 0 | reported_zero |
| Fu 2023 CISPD3 | Fu_CG | 54 | safety population/treated | TEAE leading to discontinuation of chemotherapy | 1 | 1.85 | reported |
| Jia 2025 NASCA | Jia_TG | 45 | safety population/treated | Decreased neutrophil count | 15 | 33.33 | reported |
| Jia 2025 NASCA | Jia_TG | 45 | safety population/treated | Anemia | 1 | 2.22 | reported |
| Jia 2025 NASCA | Jia_TG | 45 | safety population/treated | Decreased platelet count | 1 | 2.22 | reported |
| Jia 2025 NASCA | Jia_TG | 45 | safety population/treated | Peripheral neuropathy | 5 | 11.11 | reported |
| Jia 2025 NASCA | Jia_TG | 45 | safety population/treated | irAE any grade | 10 | 22.22 | reported |
| Jia 2025 NASCA | Jia_TG | 45 | safety population/treated | Grade >=3 irAE | 2 | 4.44 | reported |
| Jia 2025 NASCA | Jia_TG | 45 | safety population/treated | AE leading to discontinuation any medication | 15 | 33.33 | reported |
| Jia 2025 NASCA | Jia_TG | 45 | safety population/treated | Treatment-related death | 0 | 0 | reported_zero |
| Jia 2025 NASCA | Jia_CG | 45 | safety population/treated | Decreased neutrophil count | 16 | 35.56 | reported |
| Jia 2025 NASCA | Jia_CG | 45 | safety population/treated | Anemia | 1 | 2.22 | reported |
| Jia 2025 NASCA | Jia_CG | 45 | safety population/treated | Decreased platelet count | 4 | 8.89 | reported |
| Jia 2025 NASCA | Jia_CG | 45 | safety population/treated | Peripheral neuropathy | 0 | 0 | reported_zero |
| Jia 2025 NASCA | Jia_CG | 45 | safety population/treated | irAE any grade | 0 | 0 | reported_zero |
| Jia 2025 NASCA | Jia_CG | 45 | safety population/treated | Grade >=3 irAE | 0 | 0 | reported_zero |
| Jia 2025 NASCA | Jia_CG | 45 | safety population/treated | AE leading to discontinuation any medication | 10 | 22.22 | reported |
| Jia 2025 NASCA | Jia_CG | 45 | safety population/treated | Treatment-related death | 0 | 0 | reported_zero |

Safety denominators refer to the safety population/treated population. event_status distinguishes reported_zero from NR/NA.

**Appendix Table 12. Internal extraction check log and data-use warnings.**

| **Check item** | **Result** | **Details** |
| --- | --- | --- |
| Study classification | PASS with caveat | First five uploaded clinical articles correspond to NRCT/single-arm; later four randomized studies include PRINCE, PA.7, CISPD3, and NASCA. PRINCE is randomi... |
| 0 vs NA distinction | PASS | Reported zeros retained as numeric 0; unreported or not extractable values are blank with status=NR/NA_not_applicable/notes. No NR was converted to zero. |
| Fu 2023 OS/PFS HRs | CORRECTED | Uploaded article reports OS HR=1.07 (95%CI 0.69-1.68) and PFS HR=0.93 (95%CI 0.62-1.40). Older manuscript values HR 0.63/0.60 should not be used. |
| Fu response denominators | FLAGGED | Published ORR/DCR use response-evaluable denominators 44/46. ITT-conservative rows 55/55 are included for sensitivity. |
| Renouf safety denominator | FLAGGED | Control safety denominator is N=58 because 3 randomized control patients were not treated; ITT/response denominator is N=61. |
| Renouf DCR component inconsistency | FLAGGED | Use article-text DCR 84/119 and 35/61 for R. Some extracted component counts do not reconcile exactly; avoid deriving DCR from component rows. |
| Weiss denominator alignment | FLAGGED | Efficacy first-line chemo-naive treated N=12 and response-evaluable N=11; safety not cleanly separated by first-line subset. Safety rows are audit-only unles... |
| Song discontinuation | FLAGGED | Uploaded article does not give a count for AE-related discontinuation; despite older draft table, this workbook marks discontinuation as NR. |
| Stage split | PARTIAL | Chen, Cheng, Jia have LAPC/mPDAC counts; Song stage split is not extractable; PRINCE/Renouf/Morizane are metastatic-only; Fu is metastatic/recurrent. |
| CI handling | PASS | Reported CIs retained; Wilson 95% CI estimated only for missing rate CIs and marked as estimated. |
